# The Frequency of Atypical and Extreme Values for Pharyngeal Phase Swallowing Measures in Mild Parkinson Disease Compared to Healthy Aging

**DOI:** 10.1044/2021_JSLHR-21-00084

**Published:** 2021-07-27

**Authors:** Pooja Gandhi, Renata Mancopes, Danielle Sutton, Emily K. Plowman, Catriona M. Steele

**Affiliations:** a Swallowing Rehabilitation Research Laboratory, KITE Research Institute – University Health Network, Toronto, Ontario, Canada; b Rehabilitation Sciences Institute, University of Toronto, Ontario, Canada; c Aerodigestive Research Core, University of Florida, Gainesville

## Abstract

**Purpose:**

Dysphagia is thought to be prevalent and a leading cause of morbidity and mortality in people with Parkinson disease (PwPD). The aim of this study was to compare the frequencies of atypical and extreme values for measures of swallowing physiology in PwPD and in an age- and sex-matched cohort of healthy adults. Atypical and extreme values were defined, respectively, as values falling in the 25% and 5% tails of the reference distribution for healthy adults under age 60 years.

**Method:**

A standard videofluoroscopy (VF) protocol was performed in 17 adults with mild PD and 17 age- and sex-matched healthy adults using 20% w/v liquid barium ranging from thin to extremely thick consistency. Blinded VF analysis was performed according to the Analysis of Swallowing Physiology: Events, Kinematics and Timing Method. Frequencies for atypical and extreme values were tabulated by cohort and compared using odds ratios.

**Results:**

Increased frequencies of atypical values (> 25%) were seen in the PwPD for prolonged swallow reaction time, prolonged time-to-laryngeal-vestibule-closure (LVC), and poor pharyngeal constriction. However, these findings were also observed in the healthy controls. The PwPD showed significantly higher odds of atypical values for narrow upper esophageal sphincter (UES) diameter on thin liquids, a short hyoid-burst-to-UES-opening interval on extremely thick liquids, and prolonged time-to-LVC, LVC duration, and UES opening duration on multiple consistencies. The frequencies of extreme values failed to show any significant cohort differences for any parameter.

**Conclusions:**

In this study, a group of people with mild PD did not show clear evidence of swallowing impairments distinct from the changes seen in a healthy age-matched control group when odds ratios were used to compare the frequencies of atypical values between PwPD and the control group; only a few parameters showed significant differences. These were findings of significantly higher frequencies in PwPD of prolonged LVC and UES opening duration.

**Supplemental Material:**

https://doi.org/10.23641/asha.15032241

Parkinson disease (PD) is one of the most common neurological disorders internationally (Pringsheim et al., 2014). In the context of increasing life expectancies globally, a steady increase in PD is anticipated, with projections suggesting that almost 9 million people will be affected by 2030 (Dorsey et al., 2007; Pringsheim et al., 2014; Suttrup & Warnecke, 2016). Studies indicate that a majority of patients with PD develop dysphagia (swallowing impairment) during the disease course, with sex, age, and disease duration as influencing factors (Cereda et al., 2014; Kalf et al., 2012; Miller et al., 2009). Dysphagia carries a significant burden for people with PD (PwPD) as it is associated with malnutrition, aspiration pneumonia, social isolation, reduced quality of life, and mortality (Baijens & Speyer, 2009; Beyer et al., 2001; Broadfoot et al., 2019; Kalf et al., 2012; Plowman-Prine et al., 2009). In order to assess and manage PD-related dysphagia appropriately, it is imperative for clinicians to have a thorough understanding of the physiological mechanisms that contribute to impairment. Furthermore, given the known variability of deglutition and changes that occur with healthy aging, it is important for clinicians to be able to differentiate changes in swallowing that are attributable to PD from those seen in healthy aging (Kendall, 2002; Kendall & Leonard, 2001; Mancopes et al., 2021; Namasivayam-MacDonald et al., 2018; Robbins et al., 1992). The pathophysiological mechanisms underlying dysphagia in PwPD have not yet been conclusively determined.

Existing literature describing swallowing in PwPD, as compared to healthy controls, has reported reduced displacement of laryngeal and pharyngeal structures (Kim et al., 2015); compromised swallowing safety and efficiency (Nascimento et al., 2020); delayed swallow initiation and airway closure (Ellerston et al., 2016; Kim et al., 2015; Nagaya et al., 1998; Nascimento et al., 2020; Robbins et al., 1986); delayed velopharyngeal junction closure (Baijens et al., 2011); abnormal sequencing and duration of swallowing events (Nagaya et al., 1998); increased variability in swallowing pressures (Jones & Ciucci, 2016; Jones et al., 2018); and increased pharyngeal area, both at rest and at maximum constriction (Curtis et al., 2020b; Ellerston et al., 2016). The majority of these studies involved participants with moderate–severe PD, tested a limited range of bolus consistencies and/or volumes, and reported data for a limited number of swallowing parameters with varying definitions. Additionally, several of these studies reported poor inter- and intrarater reliability, particularly for measures related to velopharyngeal junction closure and upper esophageal sphincter (UES) opening (Baijens et al., 2011).

Notwithstanding these limitations, prolonged time-to-laryngeal-vestibule-closure (LVC) and reduced pharyngeal constriction emerge from the literature as commonly reported characteristics of swallowing in PwPD. However, not all studies concur in this respect. Ellerston et al. (2016) reported that delayed LVC was present on at least one thin liquid bolus in 62% of their sample of 34 PwPD, with disease severity described as “more severe,” based on a mean score of 21 (range: 9–37) on the Unified Parkinson Disease Rating Scale (UPDRS). Similarly, a recent study by Nascimento et al. (2020) reported significantly prolonged intervals between glossopharyngeal junction opening (GPJO) and LVC on 5-ml nectar-thick liquid swallows in 50 PwPD as compared to healthy controls. Disease stage in the PwPD was described as “early-intermediate” based on Hoehn and Yahr (H & Y) criteria (Hoehn & Yahr, 1967). Swallowing function was found to be unaffected by ON/OFF medication state. By contrast, Baijens et al. (2011), who used the same measures as Nascimento et al. (2020), did not find differences in time-to-LVC on 10-ml thin liquid swallows in 10 PwPD who reported swallowing complaints, compared to healthy controls. Instead, Baijens et al. (2011) reported that their participants with PD had delayed velopharyngeal junction closure, relative to GPJO. The PwPD in the Baijens et al. study were described to have mild-to-moderate disease severity based on H & Y criteria. Ellerston et al. (2016) reported that PwPD had significantly reduced pharyngeal constriction compared to healthy controls. This same observation was reported by Curtis et al. (2020b) for a sample of 40 adults with early to midstage PD. Poor pharyngeal constriction is thought to be a key predictor of pharyngeal residue in people with dysphagia (Curtis et al., 2020a; Stokely et al., 2015; Waito et al., 2018). Neither Nascimento et al. (2020) nor Baijens et al. (2011) measured pharyngeal constriction in their analyses; however, Nascimento et al. (2020) did report that residue was present either in the oral cavity and/or valleculae in at least half of their PD cohort, and in the pyriform sinuses in 8%–22% of the PD group, depending on bolus consistency. Residue was described to be more common with thicker consistencies. It is important to note that the majority of these studies reported data for fixed volumes of single consistencies. Differences in contrast media, bolus volumes, and bolus consistencies may have contributed to discrepancies in results across studies, as well as differences in the severity and duration of PD across study participants.

In order to clarify our understanding of the characteristic differences in swallowing physiology that may be expected in PwPD, it is necessary to situate measures of swallowing in PwPD in the broader context of reference values for healthy swallowing of different bolus consistencies. Recently, reference data have been published for videofluoroscopic measures of swallowing physiology in healthy adults under age 60 years, measured using the ASPEKT (Analysis of Swallowing Physiology: Events, Kinematics and Timing) Method, across the consistency range from thin to extremely thick liquids (Steele et al., 2019). We aimed to compare swallowing in PwPD to these reference data, defining values falling in the 25% tails and 5% of the reference distribution as “atypical” and “extreme,” respectively. Furthermore, because PD is more common in adults over age 65 years (Marras et al., 2018), it is also important to differentiate the characteristics of swallowing in PwPD from those seen in healthy older adults. Although our lab has collected data regarding swallowing in a cohort of healthy older adults, aged 60–82 years, collected using the ASPEKT method, to date, we have only published reference data for naturally sized sups of 20% w/v thin liquid barium for that sample (Mancopes et al., 2021). Therefore, for this study, we selected participants from our healthy data set of swallowing across the full range of liquid consistencies (thin to extremely thick) to serve as an age- and sex-matched control group for comparison to the PD cohort.

Rather than performing direct statistical comparisons of observed values between the PD and control groups, we adopted an approach similar to that used by Ellerston et al. (2016), exploring instead the frequencies of atypical and extreme values in both cohorts, relative to the available reference data for healthy younger adults (Steele et al., 2019; Steele Swallowing Lab, 2020). By definition, and as illustrated in Figure 1, the prevalence (i.e., number of participants) with values below the 25th percentile of the healthy reference distribution (equivalent to a *z* score of −0.67 on a standardized normal distribution) would not be expected to exceed 25% in a new sample of healthy adults under age 60 years, and the corresponding number of participants with values above the 75th percentile (equivalent to a *z* score of 0.67) would likewise not be expected to exceed 25%. Similarly, the prevalence of values in the 5% tails of the healthy reference distribution (i.e., either below the 5th percentile or above the 95th percentile) would not be expected to exceed 5%. In this article, we will refer to values in the 25% tails of the healthy reference distribution as “atypical” and to those in the 5% tails as “extreme” values. The premise of the analysis in this study is that when the prevalence of atypical values exceeds 25% in a new sample, or when the prevalence of extreme values exceeds 5%, one may conclude that the new sample differs in its characteristics from the published reference data. Additionally, comparisons of the prevalence of atypical and extreme values between PwPD and age- and sex-matched controls can shed light on the features of swallowing that are explained by PD as opposed to healthy aging.

**Figure 1.**
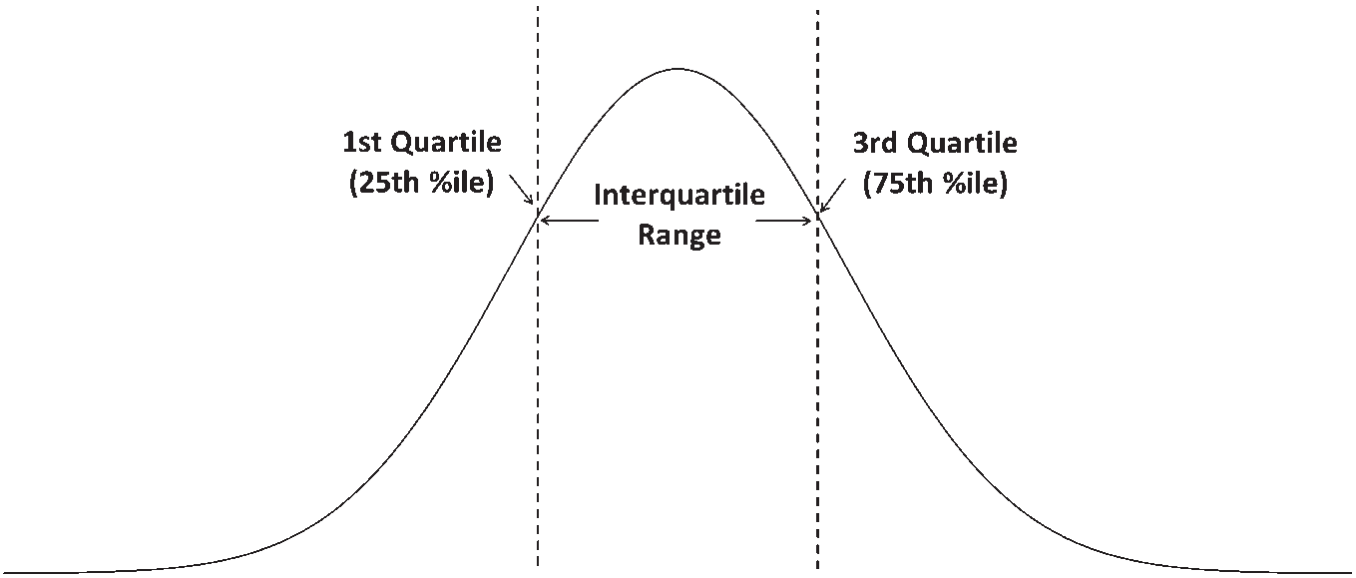
The interquartile range of a data distribution. The dotted lines show the boundaries of the interquartile range (i.e., the 25th and 75th percentiles, denoted as Q1, and Q3, respectively). In this study, swallowing measures were defined as atypical if they fell outside the interquartile range of the reference distribution (i.e., below the 25th or above the 75th percentile).

Our goal was to identify differences in swallowing physiology that may be considered characteristic of PD, through use of a standard videofluoroscopy protocol and comparison of the frequencies of typical, atypical, and extreme values in samples of PwPD and healthy older controls. Based on prior literature regarding changes in swallowing that can be expected with healthy aging (Jardine et al., 2018; Kendall & Leonard, 2001; Kendall et al., 2000; Mancopes et al., 2021; Molfenter et al., 2019; Namasivayam-MacDonald et al., 2018), we expected to find elevated frequencies (i.e., > 25% and > 5%, respectively) of atypical and extreme values in the healthy control group in the form of prolonged swallow reaction time (SRT), prolonged LVC duration, prolonged UES opening duration, and reduced pharyngeal constriction. Similarly, based on previous literature, we hypothesized that PwPD would show elevated frequencies of atypical and extreme values in the form of prolonged SRT, prolonged time-to-LVC, and reduced pharyngeal constriction. We were curious to determine, however, whether the odds of atypical values in these or other parameters would be significantly greater in the PwPD than in the age-matched healthy control group.

## Method

### Participants

A sample of adult participants with a neurologist-confirmed diagnosis of idiopathic PD was recruited from the University of Florida Center for Movement Disorders from August 2017 to March 2020. The clinical severity of the disease was scored using either the H & Y staging scale (Hoehn & Yahr, 1967) or the UPDRS. Participants were excluded if they had medical history significant for cancer, prior surgery or radiation to the speech or swallowing apparatus (other than routine dental surgery, tonsillectomy, or adenoidectomy), clinical history of dysphagia or neuromuscular disorder unrelated to the primary diagnosis, severe cognitive impairment that would impair the participant's ability to follow necessary instructions during the study procedures, significant respiratory compromise (e.g., reliance on mechanical ventilation, diaphragmatic pacer, or forced vital capacity below 65%), Type 1 diabetes, or known allergies to latex or any of the stimulus ingredients or materials used in data collection. Pregnant women were also excluded. For the purposes of comparison, data for sex- and age-matched healthy adults were extracted from a data set collected over the same time period and using the same methods at the Steele Swallowing Lab at the KITE Research Institute, Toronto Rehabilitation Institute – University Health Network. The healthy participants reported no history of swallowing complaints; difficulties with motor speech, gastroesophageal, or neurological function; sinusitis; or taste disturbance. The exclusion criteria were the same as those outlined above for the PwPD. Written informed consent was obtained from each participant prior to enrollment in the study. The study protocol received human subjects ethical approval from the local institutional review boards in Florida and Toronto, respectively. Self-report data regarding participant race and ethnicity were collected, as required by the funding agency.

### Protocol

Participants completed an intake questionnaire to confirm eligibility and document demographic information, and underwent a videofluoroscopy. The videofluoroscopy was performed at 30 frames/s and included boluses of thin, slightly thick, mildly thick, moderately thick, and extremely thick liquid as defined by the International Dysphagia Diet Standardisation Initiative framework. The barium stimuli were prepared using Bracco E-Z-Paque powdered barium in 20% w/v barium concentration and thickened with either starch- or gum-based thickening agents (Nestlé Resource ThickenUp and ThickenUp Clear, respectively). Boluses were served in separate 4-oz cups, each containing 40 ml of liquid. To maximize the ecological validity of the experiment in simulating swallowing behaviors that might occur outside an assessment context, natural sip size and three repeated boluses of each stimulus were incorporated into the protocol. Participants were instructed to take a comfortable sip from each cup for the thin, slightly and mildly thick liquids, and a comfortable spoonful using a teaspoon for the moderately and extremely thick liquids. Sip volumes were calculated based on pre- and postsip cup weights for all stimuli, taken on a digital balance. Boluses were presented in the following order:

1. Three boluses of Level 0 thin liquid;
2. Three boluses of Level 1 slightly thick liquid (gum-thickened);
3. Three boluses of Level 1 slightly thick liquid (starch-thickened);
4. Three boluses of Level 2 mildly thick liquid (gum-thickened);
5. Three boluses of Level 3 moderately thick liquid (gum-thickened);
6. Three boluses of Level 3 moderately thick liquid (starch-thickened);
7. Three boluses of Level 4 extremely thick liquid (gum-thickened).

We report the findings specific to the gum-thickened stimuli.

### Videofluoroscopy Rating

All videofluoroscopy ratings were performed in duplicate according to a standard operating procedure known as the ASPEKT Method, which has been described in previous publications (Barbon et al., 2020; Mancopes et al., 2020, 2021; Steele et al., 2019; Waito et al., 2020). Briefly, the videofluoroscopy recordings were spliced into separate clips for each bolus and randomly assigned to two trained raters. The procedure began with identification of the number of swallows per bolus and the classification of higher order swallows as either clearing swallows (of pharyngeal residue with no new material added from the mouth) or piecemeal swallows, in which additional bolus material was transferred from the mouth to the pharynx. Next, Penetration–Aspiration Scale (PAS; Rosenbek et al., 1996) ratings for each swallow were completed and LVC integrity (complete or incomplete) was identified. Frame numbers for a series of key events (from which timing measures were derived) were noted: bolus passing mandible, hyoid burst (HYB) onset, UES opening, maximum UES distension, UES closure, LVC, LVC offset, maximum pharyngeal constriction (MPC), and swallow rest. Pixel-based tracing was used to measure maximum UES opening diameter on the frame of maximum UES distension, pharyngeal area on the frames of MPC and swallow rest, and pharyngeal residue on the frame of swallow rest. All pixel-based measures were normalized to an anatomical scalar defined as the length of the C2–C4 vertebral spine (Molfenter & Steele, 2014). Discrepancies across ratings were resolved by consensus in a review session involving three experienced raters. Prior to the analysis, the data were summarized as follows: (a) The “worst” values for PAS and LVC integrity across the boluses of each consistency were captured for each participant, and (b) participant mean values were calculated across boluses, by consistency, for all other parameters. These summarized values were then coded as typical or atypical, relative to reference data published for healthy adults under age 60 years (Steele et al., 2019; Steele Swallowing Lab, 2020). Inspection of those healthy reference values shows that the strong majority of liquid boluses are swallowed in a single swallow; here, we coded situations with two swallows per bolus as atypical and situations with three or more swallows per bolus as extreme. Similarly, the healthy reference values show that PAS scores of 1 are seen in the large majority of cases. Therefore, PAS scores of 1 were coded as typical, scores of 2 were coded as atypical, and scores of 3 or higher were coded as extreme. For LVC integrity, complete closure was coded as typical, partial closure as atypical, and incomplete closure as extreme. For all other parameters, values were rounded to the closest integer value (or to the closest percent value for pixel-based measures) before classification. Atypical values were those falling below the 25th or above the 75th percentile of the healthy reference range. Atypical values were further coded as extreme if they fell in the 5% tails of the healthy reference distribution. Additional details and operational definitions regarding the ASPEKT Method can be found in Table 1.

**Table 1.** Details and definitions for the swallowing parameters rated using the ASPEKT Method (Steele et al., 2019).

Parameter	Details
Number of swallows per bolus	The rater is asked to review the entire bolus-level video clip and to document the number of swallow(s). The following components must be present in order to consider a “swallow” to have occurred: (a) at least one of laryngeal elevation, hyoid excursion, and/or pharyngeal constriction and (b) upper esophageal sphincter opening (UESO).
Penetration–aspiration	Airway invasion is rated for each and every swallow using the 8-point Penetration–Aspiration Scale (PAS; Rosenbek et al., 1996). In the case where there is more than one swallow for a bolus, the highest score seen across all swallows for that bolus is also recorded.
Laryngeal vestibule closure (LVC) integrity	The frame of LVC (or greatest approximation of LVC approximation) is reviewed, and the rater judges whether closure is complete (or incomplete). A rating of “complete” requires a seal between the epiglottis and the arytenoids, leaving no visible airspace.
Frame of hyoid burst (HYB) onset	The first anterior–superior “jump” of the hyoid that is associated with a swallow. This event has previously been referred to using the terminology *onset of maximal hyoid excursion* or *onset of the pharyngeal response* (Robbins et al., 1992).
Frame of LVC	The first frame showing maximum contact between the arytenoid process and the inferior surface of the epiglottis. In cases where there is no contact, the frame of maximum approximation of the arytenoid process to the inferior surface of the epiglottis is used.
Frame of upper esophageal sphincter opening (UESO)	The first frame where the leading edge of the bolus (or, in rare cases, air) passes through the UES. The UES is a narrow segment or region that typically lies between C4 and C6; the narrowest opening seen between C4 and C6 during a swallow is marked as the location of the sphincter (Leonard et al., 2004). In addition, recognizing that the UES moves superiorly during the swallow (Kahrilas et al., 1992), the narrowest portion may be located above C4. The superior boundary of the tracheal air column can be used as a guide to decide where the location of the UES is during pharyngeal shortening.
Frame of maximum UES distension (UESMax)	The frame where the UESO has the widest width (i.e., diameter), judged perpendicular to the cervical spine on a lateral-view fluoroscopy image.
Frame of maximum pharyngeal constriction (MPC)	The earliest frame showing maximum obliteration of the space in the pharynx. This event must occur before the upper pharynx begins to relax and before the tracheal air column begins to descend.
Frame of UES closure (UESC)	The first frame where the UES achieves closure behind the bolus tail. This does not require closure of the entire UES segment, simply closure at a single point along the segment.
Frame of LVC offset (LVCOff)	The first frame where there is visible opening (white space) of the laryngeal vestibule. This requires some separation of the tissues or of the arytenoids from the inferior surface of the epiglottis, but complete opening is not required. The leaf of the epiglottis may still be in a downward position. This event cannot be identified in cases of incomplete LVC.
Swallow rest	The terminal event of each swallow, identified as the first frame showing the pyriform sinuses at their lowest position, relative to the spine, prior to any hyoid burst or laryngeal elevation for a subsequent swallow. On the terminal swallow for a bolus, this event is further defined as occurring within 30 frames (approximately 1 s) of UESC, prior to any nonswallow events such as coughing, talking, or UES reopening.
Swallow reaction time	The time interval between BPM and HYB
hyoid-burst-to-UES-opening	The time interval between HYB and UESO
UES opening duration	The time interval between UESO and UESC
time-to-LVC	The time interval between HYB and the frame of LVC
LVC duration	The time interval between the frames of LVC and LVCOff; this parameter is only recorded in cases where LVC integrity is complete.

*Note.* BPM = the time interval between the frame of bolus passing mandible (BPM) and HYB.

### Statistical Analysis

Statistical analyses were conducted using SPSS 27.0. Interrater reliability was calculated on original ratings (prior to discrepancy resolution) using percent absolute agreement for categorical parameters and intraclass correlations for continuous parameters. Frequency tables for the number of participants showing atypical values were compiled for each parameter by cohort and liquid consistency. Whenever the number of participants displaying atypical values within a cohort exceeded 25%, this was noted as a finding of interest. Similarly, frequency tables for the number of participants showing extreme values were compiled, with frequencies > 5% being flagged as a finding of interest. Finally, cohort differences in the frequencies of atypical and extreme values were explored using odds ratios (ORs). In cases where there were zero cell counts in one cohort, ORs were estimated using the Haldane–Anscombe correction (Lawson, 2004). ORs > 1.0 and with confidence intervals that did not cross a value of 1.0 were considered significant.

## Results

### Participants

As shown in Table 2, the study sample included 34 participants: 17 with PD and 17 age- and sex-matched healthy controls. The sex distribution was skewed in favor of males, with each group containing 13 males and four females. Median age was 69 years (range: 53–85 years) in the PD cohort and 68 years (range: 53–82 years) in the healthy control group. In the PD cohort, two participants reported their race as African American and ethnicity as non-Hispanic, with the remaining 15 reporting their race as White, with one individual reporting ethnicity as Hispanic. The healthy control participants were drawn from the same data set used previously by Mancopes et al. (2021), based on sex and age match with the individuals with PD. Among the healthy controls, 13 individuals reported race as White, three as Asian, and one as American Indian; none of the control participants reported their ethnicity as Hispanic. The mean PD severity score as per the H & Y scale (Hoehn & Yahr, 1967) was 2, suggesting mild disease. All participants with PD were assessed in the medication “on” state.

**Table 2.** Demographic characteristics of the study sample.

Parameter	PwPD	Healthy control group
Number of participants	17	17
Median age (IQR)	69 (67–72)	68 (67–73)
Sex (% male)	13 (76.5%)	13 (76.5%)
Months since diagnosis (range)	45 (18–84)	
Average H & Y Scale score (*n* = 13)	2	
Average UPDRS score (*n* = 5)	22	
Receiving L-Dopa	17 (100%)	
Receiving DBS	1 (5.8%)	

*Note.* PwPD = people with Parkinson disease; IQR = Interquartile range; H & Y = Hoehn and Yahr; UPDRS = Unified Parkinson Disease Rating Scale; L-Dopa = Levodopa; DBS = deep-brain stimulation.

### Interrater Agreement

Interrater reliability was calculated on raw rating scores, prior to consensus resolution. The results are summarized in Table 3 using percent absolute agreement for categorical parameters and intraclass correlations for continuous parameters. Overall, results suggested excellent agreement for frame identification, and for ratings of binary, categorical, and continuous videofluoroscopy measures. Agreement for pixel-based measures of residue was good (valleculae) to moderate (pyriform sinus residue), while initial measures of UES diameter showed poor agreement. As per protocol, all discrepancies were reviewed and resolved in a consensus meeting.

**Table 3.** Preconsensus interrater agreement for swallowing parameters expressed as percentage absolute agreement and intraclass correlation coefficients.

Categorical parameters	Percentage absolute agreement	
Penetration–Aspiration Scale score	99.70%	
Laryngeal vestibule closure integrity	100.00%	
**Continuous parameters**	**ICC** *	**95% CI**
First frame of bolus passing mandible	.955	[0.945, 0.964]
First frame of hyoid burst	.973	[0.966, 0.978]
First frame of laryngeal vestibule closure	.982	[0.978, 0.986]
First frame of maximum pharyngeal closure	.989	[0.987, 0.991]
Swallow rest frame	.971	[0.964, 0.976]
First frame of upper esophageal sphincter opening	.989	[0.987, 0.991]
First frame of upper esophageal sphincter closure	.985	[0.982, 0.988]
Frame of upper esophageal sphincter maximum diameter	.984	[0.981, 0.987]
Vallecular residue	.722	[0.664, 0.770]
Pyriform sinus residue	.754	[0.701, 0.798]

*Note.* CI = confidence interval.

* ICC (intraclass coefficient) values less than .5 are indicative of poor reliability, values between .5 and .75 indicate moderate reliability, values between .75 and .9 indicate good reliability, and values greater than .90 indicate excellent reliability.

### Descriptive Statistics and Frequencies

Descriptive statistics for all parameters can be found by cohort and bolus consistency in Supplemental Material S1. The majority (i.e., 87%) of the 510 boluses administered in this study were swallowed in a single swallow. Secondary swallows were seen on 58 boluses, with 90% of these classified as piecemeal swallows, rather than clearing swallows. Tertiary and quaternary swallows were seen on 19 and two boluses, respectively. A single tertiary swallow was classified as clearing swallow, with all remaining third- and fourth-order swallows classified as piecemeal. Participant-level frequency data for atypical values falling beyond the 25th and 75th percentiles of the healthy reference distribution will be presented below, and followed by frequency data for extreme values.

### Atypical Values

Table 4 shows the frequency (*n,* %) of atypical values for each ASPEKT parameter in each cohort by bolus consistency. The frequencies of atypical values are compared between the PwPD and healthy control cohorts using ORs in Table 5.

**Table 4.** Frequencies (participants per cohort) for atypical^a^ videofluoroscopic values by bolus consistency.

Parameter	Direction of atypical^a^ value	IDDSI level	Atypical			
			PwPD	Healthy controls		
			*n*	%	*n*	%
Sip volume	Smaller	0 (Thin)	5	29	2	12
		1 (Slightly thick)	3	18	1	6
		2 (Mildly thick)	4	24	1	6
		3 (Moderately thick)	1	6	3	18
		4 (Extremely thick)	1	6	2	12
	Larger	0 (Thin)	4	24	9	53
		1 (Slightly thick)	8	47	9	53
		2 (Mildly thick)	10	59	8	47
		3 (Moderately thick)	7	41	7	41
		4 (Extremely thick)	6	35	6	35
Number of swallows	Larger	0 (Thin)	6	35	3	18
		1 (Slightly thick)	3	18	1	6
		2 (Mildly thick)	2	12	1	6
		3 (Moderately thick)	2	12	1	6
		4 (Extremely thick)	2	12	1	6
Penetration–aspiration on initial swallow	Worse (higher score)	0 (Thin)	5	29	5	29
		1 (Slightly thick)	4	24	5	29
		2 (Mildly thick)	2	12	2	12
		3 (Moderately thick)	1	6	1	6
		4 (Extremely thick)	1	6	0	0
Maximum penetration–aspiration for bolus	Worse (higher score)	0 (Thin)	5	29	5	29
		1 (Slightly thick)	4	24	5	29
		2 (Mildly thick)	3	18	2	12
		3 (Moderately thick)	1	6	1	6
		4 (Extremely thick)	2	12	0	0
Swallow reaction time	Shorter	0 (Thin)	0	0	2	12
		1 (Slightly thick)	1	6	2	12
		2 (Mildly thick)	0	0	1	6
		3 (Moderately thick)	0	0	0	0
		4 (Extremely thick)	1	6	1	6
	Longer	0 (Thin)	12	71	11	65
		1 (Slightly thick)	8	47	7	41
		2 (Mildly thick)	7	41	8	47
		3 (Moderately thick)	6	35	8	47
		4 (Extremely thick)	8	47	9	53
Hyoid-burst-to-UES-opening interval	Shorter	0 (Thin)	1	6	3	18
		1 (Slightly thick)	3	18	6	35
		2 (Mildly thick)	3	18	4	24
		3 (Moderately thick)	2	12	1	6
		4 (Extremely thick)	9	53	2	12
	Longer	0 (Thin)	6	35	2	12
		1 (Slightly thick)	5	29	3	18
		2 (Mildly thick)	3	18	4	24
		3 (Moderately thick)	4	24	5	29
		4 (Extremely thick)	3	18	4	24
UES opening duration	Shorter	0 (Thin)	2	12	2	12
		1 (Slightly thick)	1	6	2	12
		2 (Mildly thick)	3	18	3	18
		3 (Moderately thick)	3	18	3	18
		4 (Extremely thick)	3	18	2	12
	Longer	0 (Thin)	10	59	6	35
		1 (Slightly thick)	13	81	7	41
		2 (Mildly thick)	6	35	3	18
		3 (Moderately thick)	9	53	3	18
		4 (Extremely thick)	8	47	3	18
Time-to-LVC	Shorter	0 (Thin)	3	18	3	18
		1 (Slightly thick)	2	12	2	12
		2 (Mildly thick)	3	18	4	24
		3 (Moderately thick)	2	12	0	0
		4 (Extremely thick)	1	6	0	0
	Longer	0 (Thin)	6	35	2	12
		1 (Slightly thick)	8	47	1	6
		2 (Mildly thick)	9	53	3	18
		3 (Moderately thick)	8	47	4	24
		4 (Extremely thick)	10	59	7	41
LVC duration	Shorter	0 (Thin)	1	6	2	12
		1 (Slightly thick)	1	6	1	6
		2 (Mildly thick)	1	6	1	6
		3 (Moderately thick)	2	12	0	0
		4 (Extremely thick)	3	18	2	12
	Longer	0 (Thin)	9	56	8	47
		1 (Slightly thick)	12	71	6	35
		2 (Mildly thick)	12	71	4	24
		3 (Moderately thick)	8	47	4	24
		4 (Extremely thick)	7	41	4	24
UES diameter	Narrower	0 (Thin)	7	44	0	0
		1 (Slightly thick)	5	31	0	0
		2 (Mildly thick)	2	13	1	6
		3 (Moderately thick)	3	19	0	0
		4 (Extremely thick)	3	19	0	0
	Wider	0 (Thin)	1	6	6	35
		1 (Slightly thick)	5	31	7	41
		2 (Mildly thick)	2	13	10	59
		3 (Moderately thick)	3	19	7	41
		4 (Extremely thick)	6	38	7	41
Pharyngeal area at maximum constriction	Larger (i.e., poorer constriction)	0 (Thin)	5	31	6	35
		1 (Slightly thick)	7	44	8	47
		2 (Mildly thick)	6	38	6	35
		3 (Moderately thick)	12	71	9	53
		4 (Extremely thick)	10	59	8	47
Vallecular residue	Larger	0 (Thin)	4	25	7	41
		1 (Slightly thick)	5	31	8	47
		2 (Mildly thick)	4	24	4	24
		3 (Moderately thick)	3	18	5	29
		4 (Extremely thick)	4	24	4	24
Pyriform sinus residue	Larger	0 (Thin)	2	13	4	24
		1 (Slightly thick)	2	13	4	24
		2 (Mildly thick)	2	13	5	29
		3 (Moderately thick)	0	0	4	24
		4 (Extremely thick)	1	6	3	18

*Note.* IDDSI = International Dysphagia Diet Standardisation Initiative; PwPD = people with Parkinson disease; UES = upper esophageal sphincter; LVC = laryngeal vestibule closure.

a Atypical = either below the 25th percentile or above the 75th percentile values from the healthy reference distribution.

**Table 5.** Odd ratios for atypical* videofluoroscopic values in people with Parkinson disease and healthy age-matched controls by bolus consistency.

Parameter	Direction	IDDSI level	Atypical^a^ values	
			Odds ratio PD vs. healthy	95% CI
Sip volume	Smaller	0 (Thin)	3.13	[0.51, 19.04]
		1 (Slightly thick)	3.43	[0.32, 36.83]
		2 (Mildly thick)	4.92	[0.49, 49.61]
		3 (Moderately thick)	0.29	[0.03, 3.13]
		4 (Extremely thick)	0.47	[0.04, 5.72]
	Larger	0 (Thin)	0.27	[0.06, 1.19]
		1 (Slightly thick)	0.79	[0.21, 3.04]
		2 (Mildly thick)	1.61	[0.41, 6.24]
		3 (Moderately thick)	1.00	[0.26, 3.92]
		4 (Extremely thick)	1.00	[0.24, 4.08]
Number of swallows	Larger	0 (Thin)	2.55	[0.52, 12.55]
		1 (Slightly thick)	3.43	[0.32, 36.83]
		2 (Mildly thick)	2.13	[0.17, 26.03]
		3 (Moderately thick)	2.13	[0.17, 26.03]
		4 (Extremely thick)	2.13	[0.17, 26.03]
Penetration–aspiration on initial swallow	Worse (higher score)	0 (Thin)	1.00	[0.23, 4.37]
		1 (Slightly thick)	0.74	[0.16, 3.41]
		2 (Mildly thick)	1.00	[0.12, 8.06]
		3 (Moderately thick)	1.00	[0.06, 17.41]
		4 (Extremely thick)	2.13^a^	[0.18, 26.03]
Maximum penetration–aspiration for bolus	Worse (higher score)	0 (Thin)	1.00	[0.23, 4.37]
		1 (Slightly thick)	0.74	[0.16, 3.41]
		2 (Mildly thick)	1.61	[0.23, 11.09]
		3 (Moderately thick)	1.00	[0.06, 17.41]
		4 (Extremely thick)	3.43^a^	[0.32, 36.83]
Swallow reaction time	Shorter	0 (Thin)	0.29^a^	[0.03, 3.13]
		1 (Slightly thick)	0.47	[0.04, 5.72]
		2 (Mildly thick)	0.47^a^	[0.04, 5.21]
		3 (Moderately thick)	1.00^a^	[0.06, 17.41]
		4 (Extremely thick)	1.00	[0.06, 17.41]
	Longer	0 (Thin)	1.31	[0.31, 5.53]
		1 (Slightly thick)	1.27	[0.33, 4.93]
		2 (Mildly thick)	0.79	[0.20, 3.06]
		3 (Moderately thick)	0.61	[0.15, 2.43]
		4 (Extremely thick)	0.79	[0.21, 3.04]
Hyoid-burst-to-UES-opening interval	Shorter	0 (Thin)	0.29	[0.03, 3.13]
		1 (Slightly thick)	0.39	[0.08, 1.94]
		2 (Mildly thick)	0.70	[0.13, 3.72]
		3 (Moderately thick)	2.13	[0.17, 26.03]
		4 (Extremely thick)	**8.44**	**[1.46, 48.85]**
	Longer	0 (Thin)	4.09	[0.69, 24.24]
		1 (Slightly thick)	1.94	[0.38, 9.88]
		2 (Mildly thick)	0.70	[0.13, 3.72]
		3 (Moderately thick)	0.74	[0.16, 3.41]
		4 (Extremely thick)	0.70	[0.13, 3.72]
UES opening duration	Shorter	0 (Thin)	1.00	[0.12, 8.06]
		1 (Slightly thick)	0.50	[0.04, 6.12]
		2 (Mildly thick)	1.00	[0.17, 5.83]
		3 (Moderately thick)	1.00	[0.17, 5.83]
		4 (Extremely thick)	1.61	[0.23, 11.09]
	Longer	0 (Thin)	2.62	[0.65, 10.48]
		1 (Slightly thick)	**6.19**	**[1.27, 30.17]**
		2 (Mildly thick)	2.55	[0.52, 12.55]
		3 (Moderately thick)	**5.25**	**[1.09, 25.21]**
		4 (Extremely thick)	4.15	[0.86, 19.92]
Time-to-LVC	Shorter	0 (Thin)	1.00	[0.17, 5.83]
		1 (Slightly thick)	1.00	[0.12, 8.06]
		2 (Mildly thick)	0.70	[0.13, 3.72]
		3 (Moderately thick)	2.13^a^	[0.18, 25.78]
		4 (Extremely thick)	2.13^a^	[0.18, 25.78]
	Longer	0 (Thin)	4.09	[0.69, 24.24]
		1 (Slightly thick)	**14.22**	**[1.52, 132.73]**
		2 (Mildly thick)	**5.25**	**[1.09, 25.21]**
		3 (Moderately thick)	2.89	[0.66, 12.57]
		4 (Extremely thick)	2.04	[0.52, 8.00]
LVC duration	Shorter	0 (Thin)	0.50	[0.04, 6.12]
		1 (Slightly thick)	1.00	[0.06–17.41]
		2 (Mildly thick)	1.00	[0.06–17.41]
		3 (Moderately thick)	0.47	[0.32, 0.68]
		4 (Extremely thick)	1.61	[0.23, 11.09]
	Longer	0 (Thin)	1.45	[0.37, 5.7]
		1 (Slightly thick)	**4.40**	**[1.04, 18.60]**
		2 (Mildly thick)	**7.80**	**[1.69, 36.06]**
		3 (Moderately thick)	2.89	[0.66, 12.57]
		4 (Extremely thick)	2.28	[0.52, 9.99]
UES diameter	Narrower	0 (Thin)	**14.22** ^**b**^	**[1.52, 132.74]**
		1 (Slightly thick)	8.73^b^	[0.92, 82.96]
		2 (Mildly thick)	2.46	[0.2, 30.28]
		3 (Moderately thick)	4.92^b^	[0.49, 49.61]
		4 (Extremely thick)	4.92^b^	[0.49, 49.61]
	Wider	0 (Thin)	0.12	[0.01, 1.17]
		1 (Slightly thick)	0.65	[0.16, 2.72]
		2 (Mildly thick)	0.11	[0.02, 0.64]
		3 (Moderately thick)	0.33	[0.07, 1.61]
		4 (Extremely thick)	0.86	[0.21, 3.47]
Pharyngeal area at maximum constriction	Larger (i.e., poorer constriction)	0 (Thin)	0.83	[0.20, 3.56]
		1 (Slightly thick)	0.88	[0.22, 3.45]
		2 (Mildly thick)	1.10	[0.27, 4.55]
		3 (Moderately thick)	2.13	[0.52, 8.76]
		4 (Extremely thick)	1.61	[0.41, 6.24]
Vallecular residue	Larger	0 (Thin)	0.48	[0.11, 2.11]
		1 (Slightly thick)	0.51	[0.12, 2.12]
		2 (Mildly thick)	1.00	[0.2, 4.88]
		3 (Moderately thick)	0.51	[0.1, 2.61]
		4 (Extremely thick)	1.00	[0.2, 4.88]
Pyriform sinus residue	Larger	0 (Thin)	0.50	[0.08, 3.22]
		1 (Slightly thick)	0.50	[0.08, 3.22]
		2 (Mildly thick)	0.34	[0.06, 2.1]
		3 (Moderately thick)	0.15^b^	[0.02, 1.46]
		4 (Extremely thick)	0.31	[0.03, 3.35]

*Note.* Bold values indicate statistically significant findings with odds ratios where the lower 95% confidence interval limit is greater than 1.0. IDDSI = International Dysphagia Diet Standardisation Initiative; PD = Parkinson disease; CI = confidence interval; UES = upper esophageal sphincter; LVC = laryngeal vestibule closure.

a Atypical = either below the 25th percentile or above the 75th percentile values from the healthy reference distribution.

b Estimated using Haldane–Anscombe correction.

#### Sip Volume and Number of Swallows

Smaller-than-typical sip size was seen on thin liquids in > 25% of the PwPD. Conversely, larger-than-typical sip size was seen in > 25% of the PwPD on all thicker consistencies and in > 25% of the healthy control group on all consistencies. Multiple swallows per bolus were seen in > 25% of the PwPD on thin liquids but in < 25% of the healthy control group. On thicker consistencies, neither cohort showed elevated frequencies of a multiple-swallows-per-bolus pattern. When the frequencies of atypical values were compared between the two cohorts, there were no significant differences in the odds of atypical sip size (either small or large) or of multiple swallows per bolus.

#### Penetration–Aspiration and LVC Integrity

A > 25% prevalence of atypical PAS scores (i.e., ≥ 2) was seen in the PwPD on thin liquids and on thin and slightly thick liquids in the healthy control group. When the range of individual scores was inspected, three participants in the PD cohort obtained a PAS score of 2, one participant obtained a score of 5, and one participant obtained a score of 8. In the healthy control group, four participants obtained a PAS score of 2 and one obtained a score of 5. These frequencies held true, both when considering the worst PAS scores seen on the initial swallows for each bolus across the boluses of each consistencies and when tabulating the worst maximum PAS scores seen across all swallows for each bolus. There were no significant cohort differences in the odds of PAS scores ≥ 2. LVC was judged to be complete on all boluses in both groups.

#### Timing Measures

Both the PwPD and healthy control groups displayed > 25% prevalence of atypical prolonged SRT on all consistencies, with no significant group differences in odds. The HYB-to-UES-opening interval fell within the typical reference range for both groups for most consistencies; exceptions to this observation included (a) increased prevalence (35%) of a shortened HYB to UES opening (UESO) interval on slightly thick liquids in the healthy controls, (b) dramatically increased prevalence (53%) of short HYB-UESO on extremely thick liquids in the PwPD, (c) increased prevalence of prolonged HYB-UESO on thin (35%) and slightly thick (29%) liquids in the PwPD, and (d) increased prevalence (29%) of prolonged HYB-UESO on moderately thick liquids in the healthy controls. Among these observations, only the increased frequency of short HYB-UESO on extremely thick liquids showed a significant cohort difference, with 8.44-fold higher odds in the PwPD.

Atypical prolonged UES opening duration was seen in > 25% of the PwPD on all consistencies and also in > 25% of healthy controls on thin and slightly thick liquids. When group frequencies were compared, the odds of prolonged UES opening were significantly higher (≥ fivefold) in the PwPD on the slightly thick and moderately thick liquids. Two- to fourfold increased odds of long UES opening duration were also seen on the other consistencies in the PwPD; however, the lower boundaries of the 95% confidence intervals fell below 1, thereby bringing the significance of these ratios into question.

Atypical prolonged time-to-LVC was seen in > 25% of the PD participants on all consistencies and in > 25% of the healthy control participants on extremely thick liquids. Significant cohort differences were found, with the ORs for prolonged time-to-LVC being 14-fold and fivefold higher in the PwPD, on slightly and mildly thick liquids, respectively. Atypical prolonged LVC duration was seen in > 25% of the PD group on all consistencies and in > 25% of the healthy controls on thin and slightly thick liquids. The PwPD were significantly more likely to show prolonged LVC duration on the slightly and mildly thick liquids with at least fourfold higher odds.

#### UES Opening Diameter

Atypical narrow UES opening diameter was seen in > 25% of the PD group on thin liquids. Interestingly, for slightly thick liquids, the PwPD showed increased frequencies > 25% both of atypical narrow UES opening and also of atypical wide UES opening. Atypical wide UES opening diameter was seen in > 25% the healthy cohort on all consistencies and in the PD cohort on extremely thick liquids. The odds of atypical narrow UES opening diameter were 14-fold higher in the PwPD while the odds or atypical wide UES opening diameter did not significantly different between cohorts.

#### Pharyngeal Area at Maximum Constriction

The frequency of poor pharyngeal constriction, as shown by atypical large pharyngeal area at maximum constriction, exceeded 25% in both groups on all consistencies. Group frequencies did not differ significantly.

#### Vallecular and Pyriform Sinus Residue

Vallecular residue above the 75th percentile of the reference distribution was seen in > 25% of the PD cohort for slightly thick liquids, and on thin, slightly and mildly thick liquids in the healthy control group. Frequencies did not differ significantly between cohorts. Pyriform sinus residue above the 75th percentile threshold was seen in > 25% of participants in > 25% of the healthy cohort on mildly thick liquids. Again, group frequencies were not significantly different.

### Extreme Values

Table 6 shows the frequency (*n,* %) of atypical values for each ASPEKT parameter in each cohort by bolus consistency. The frequencies of atypical values are compared between the PwPD and healthy control cohorts using ORs in Table 7.

**Table 6.** Frequencies (participants per cohort) for extreme^a^ videofluoroscopic values by bolus consistency.

Parameter	Direction of extreme^a^ value	IDDSI level	PwPD		Healthy controls	
			*n*	%	*n*	%
Sip volume	Smaller	0 (Thin)	2	12	0	0
		1 (Slightly thick)	0	0	0	0
		2 (Mildly thick)	1	6	0	0
		3 (Moderately thick)	0	0	1	6
		4 (Extremely thick)	1	6	1	6
	Larger	0 (Thin)	0	0	2	12
		1 (Slightly thick)	2	12	2	12
		2 (Mildly thick)	2	12	3	18
		3 (Moderately thick)	0	0	0	0
		4 (Extremely thick)	1	6	1	6
Number of swallows	Larger	0 (Thin)	1	6	1	6
		1 (Slightly thick)	1	6	1	6
		2 (Mildly thick)	0	0	1	6
		3 (Moderately thick)	0	0	1	6
		4 (Extremely thick)	0	0	1	6
Penetration–aspiration on initial swallow	Worse (higher score)	0 (Thin)	2	12	1	6
		1 (Slightly thick)	1	6	0	0
		2 (Mildly thick)	0	0	0	0
		3 (Moderately thick)	0	0	0	0
		4 (Extremely thick)	1	6	0	0
Maximum penetration–aspiration for bolus	Worse (higher score)	0 (Thin)	2	12	1	6
		1 (Slightly thick)	1	6	0	0
		2 (Mildly thick)	1	6	0	0
		3 (Moderately thick)	0	0	0	0
		4 (Extremely thick)	1	6	0	0
Swallow reaction time	Shorter	0 (Thin)	1	6	0	0
		1 (Slightly thick)	1	6	2	12
		2 (Mildly thick)	0	0	0	0
		3 (Moderately thick)	0	0	0	0
		4 (Extremely thick)	0	0	1	6
	Longer	0 (Thin)	2	12	1	6
		1 (Slightly thick)	2	12	1	6
		2 (Mildly thick)	4	24	1	6
		3 (Moderately thick)	3	18	0	0
		4 (Extremely thick)	0	0	0	0
Hyoid-burst-to-UES-opening interval	Shorter	0 (Thin)	0	0	1	6
		1 (Slightly thick)	1	6	2	12
		2 (Mildly thick)	0	0	1	6
		3 (Moderately thick)	0	0	0	0
		4 (Extremely thick)	1	6	0	0
	Longer	0 (Thin)	0	0	0	0
		1 (Slightly thick)	0	0	0	0
		2 (Mildly thick)	2	12	2	12
		3 (Moderately thick)	1	6	3	18
		4 (Extremely thick)	0	0	1	6
UES opening duration	Shorter	0 (Thin)	2	12	1	6
		1 (Slightly thick)	0	0	0	0
		2 (Mildly thick)	1	6	0	0
		3 (Moderately thick)	1	6	1	6
		4 (Extremely thick)	0	0	1	6
	Longer	0 (Thin)	5	29	2	12
		1 (Slightly thick)	2	12	3	18
		2 (Mildly thick)	0	0	2	12
		3 (Moderately thick)	2	12	0	0
		4 (Extremely thick)	1	6	1	6
Time-to-LVC	Shorter	0 (Thin)	1	6	2	12
		1 (Slightly thick)	1	6	0	0
		2 (Mildly thick)	2	12	1	6
		3 (Moderately thick)	0	0	0	0
		4 (Extremely thick)	0	0	0	0
	Longer	0 (Thin)	1	6	1	6
		1 (Slightly thick)	0	0	1	6
		2 (Mildly thick)	2	12	1	6
		3 (Moderately thick)	2	12	2	12
		4 (Extremely thick)	0	0	0	0
LVC duration	Shorter	0 (Thin)	1	6	0	0
		1 (Slightly thick)	1	6	0	0
		2 (Mildly thick)	1	6	0	0
		3 (Moderately thick)	2	12	0	0
		4 (Extremely thick)	1	6	0	0
	Longer	0 (Thin)	3	19	3	18
		1 (Slightly thick)	3	18	2	12
		2 (Mildly thick)	3	18	2	12
		3 (Moderately thick)	4	24	1	6
		4 (Extremely thick)	3	18	1	6
UES diameter	Narrower	0 (Thin)	2	12	0	0
		1 (Slightly thick)	1	6	0	0
		2 (Mildly thick)	0	0	0	0
		3 (Moderately thick)	1	6	0	0
		4 (Extremely thick)	0	0	0	0
	Wider	0 (Thin)	0	0	1	6
		1 (Slightly thick)	0	0	4	24
		2 (Mildly thick)	1	7	4	24
		3 (Moderately thick)	0	0	0	0
		4 (Extremely thick)	2	12	2	12
Pharyngeal area at maximum constriction	Larger (i.e., poorer constriction)	0 (Thin)	4	25	2	12
		1 (Slightly thick)	4	25	4	24
		2 (Mildly thick)	3	19	3	18
		3 (Moderately thick)	5	29	1	6
		4 (Extremely thick)	5	29	2	12
Vallecular residue	Larger	0 (Thin)	0	0	0	0
		1 (Slightly thick)	1	6	1	6
		2 (Mildly thick)	1	6	1	6
		3 (Moderately thick)	0	0	0	0
		4 (Extremely thick)	1	6	0	0
Pyriform sinus residue	Larger	0 (Thin)	1	7	2	12
		1 (Slightly thick)	2	12	2	12
		2 (Mildly thick)	1	6	0	0
		3 (Moderately thick)	0	0	1	6
		4 (Extremely thick)	0	0	1	6

*Note.* IDDSI = International Dysphagia Diet Standardisation Initiative; PwPD = people with Parkinson disease; UES = upper esophageal sphincter; LVC = laryngeal vestibule closure.

a Extreme = either below the 5th percentile or above the 95th percentile values from the healthy reference distribution.

**Table 7.** Odd ratios for extreme^a^ videofluoroscopic values in people with Parkinson disease and healthy age-matched controls by bolus consistency.

Parameter	Direction	IDDSI level	Extreme^a^ values	
			Odds ratio PD vs. healthy	95% CI
Sip volume	Smaller	0 (Thin)	3.43^b^	[0.32, 36.83]
		1 (Slightly thick)	1.00^b^	[0.06, 17.41]
		2 (Mildly thick)	2.13^b^	[0.18, 26.03]
		3 (Moderately thick)	0.47^b^	[0.04, 5.72]
		4 (Extremely thick)	1.00	[0.06, 17.41]
	Larger	0 (Thin)	0.29	[0.03, 3.13]
		1 (Slightly thick)	1.00	[0.06, 17.41]
		2 (Mildly thick)	0.62	[0.09, 4.29]
		3 (Moderately thick)	1.00^b^	[0.06, 17.41]
		4 (Extremely thick)	1.00	[0.06, 17.41]
Number of swallows	Larger	0 (Thin)	1.00	[0.06, 17.41]
		1 (Slightly thick)	1.00	[0.06, 17.41]
		2 (Mildly thick)	0.47^b^	[0.04, 5.72]
		3 (Moderately thick)	0.47^b^	[0.04, 5.72]
		4 (Extremely thick)	0.47^b^	[0.04, 5.72]
Penetration–aspiration on initial swallow	Worse (higher score)	0 (Thin)	2.13	[0.17, 26.03]
		1 (Slightly thick)	2.13^b^	[0.17, 26.03]
		2 (Mildly thick)	1.00^b^	[0.06, 17.41]
		3 (Moderately thick)	1.00^b^	[0.06, 17.41]
		4 (Extremely thick)	2.13^b^	[0.17, 26.03]
Maximum penetration–aspiration for bolus	Worse (higher score)	0 (Thin)	2.13	[0.17, 26.03]
		1 (Slightly thick)	2.13^b^	[0.17, 26.03]
		2 (Mildly thick)	2.13^b^	[0.17, 26.03]
		3 (Moderately thick)	1.00^b^	[0.06, 17.41]
		4 (Extremely thick)	2.13^b^	[0.17, 26.03]
Swallow reaction time	Shorter	0 (Thin)	2.13^b^	[0.17, 26.03]
		1 (Slightly thick)	1.00^b^	[0.06, 17.41]
		2 (Mildly thick)	1.00^b^	[0.06, 17.41]
		3 (Moderately thick)	1.00^b^	[0.06, 17.41]
		4 (Extremely thick)	0.47^b^	[0.04, 5.72]
	Longer	0 (Thin)	2.13	[0.17, 26.03]
		1 (Slightly thick)	2.13	[0.17, 26.03]
		2 (Mildly thick)	4.92	[0.49, 49.61]
		3 (Moderately thick)	4.92^b^	[0.49, 49.61]
		4 (Extremely thick)	1.00^b^	[0.06, 17.41]
Hyoid-burst-to-UES-opening interval	Shorter	0 (Thin)	0.47^b^	[0.04, 5.72]
		1 (Slightly thick)	0.47	[0.04, 5.72]
		2 (Mildly thick)	0.47^b^	[0.04, 5.72]
		3 (Moderately thick)	1.00^b^	[0.06, 17.41]
		4 (Extremely thick)	2.13^b^	[0.17, 26.03]
	Longer	0 (Thin)	1.00^b^	[0.06, 17.41]
		1 (Slightly thick)	1.00^b^	[0.06, 17.41]
		2 (Mildly thick)	1.00	[0.12, 8.06]
		3 (Moderately thick)	0.29	[0.03, 3.13]
		4 (Extremely thick)	0.47^b^	[0.04, 5.72]
UES opening duration	Shorter	0 (Thin)	2.13	[0.32, 36.83]
		1 (Slightly thick)	1.00^b^	[0.06, 17.41]
		2 (Mildly thick)	2.13^b^	[0.17, 26.03]
		3 (Moderately thick)	1.00	[0.06, 17.41]
		4 (Extremely thick)	0.47^b^	[0.04, 5.72]
	Longer	0 (Thin)	3.13	[0.51, 19.04]
		1 (Slightly thick)	0.67	[0.1, 4.62]
		2 (Mildly thick)	0.29	[0.03, 3.13]
		3 (Moderately thick)	3.43^b^	[0.32, 36.83]
		4 (Extremely thick)	1.00	[0.06, 17.41]
Time-to-LVC	Shorter	0 (Thin)	0.47	[0.04, 5.72]
		1 (Slightly thick)	2.13^b^	[0.17, 26.03]
		2 (Mildly thick)	2.13	[0.17, 26.03]
		3 (Moderately thick)	1.00^b^	[0.06, 17.41]
		4 (Extremely thick)	1.00^b^	[0.06, 17.41]
	Longer	0 (Thin)	1.00	[0.06, 17.41]
		1 (Slightly thick)	0.47^b^	[0.04, 5.72]
		2 (Mildly thick)	2.13	[0.17, 26.03]
		3 (Moderately thick)	1.00	[0.12, 8.06]
		4 (Extremely thick)	1.00^b^	[0.06, 17.41]
LVC duration	Shorter	0 (Thin)	2.13^b^	[0.17, 26.03]
		1 (Slightly thick)	2.13^b^	[0.17, 26.03]
		2 (Mildly thick)	2.13^b^	[0.17, 26.03]
		3 (Moderately thick)	2.13^b^	[0.17, 26.03]
		4 (Extremely thick)	2.13^b^	[0.17, 26.03]
	Longer	0 (Thin)	1.08	[0.18, 6.32]
		1 (Slightly thick)	1.61	[0.23, 11.09]
		2 (Mildly thick)	1.61	[0.23, 11.09]
		3 (Moderately thick)	4.92	[0.49, 49.61]
		4 (Extremely thick)	3.43	[0.32, 36.83]
UES diameter	Narrower	0 (Thin)	3.43a	[0.32, 36.83]
		1 (Slightly thick)	2.13a	[0.18, 26.03]
		2 (Mildly thick)	1.00^b^	[0.06, 17.41]
		3 (Moderately thick)	2.13^b^	[0.18, 26.03]
		4 (Extremely thick)	1.00^b^	[0.06, 17.41]
	Wider	0 (Thin)	0.47^b^	[0.04, 5.72]
		1 (Slightly thick)	0.15^b^	[0.02, 1.46]
		2 (Mildly thick)	0.23	[0.02, 2.36]
		3 (Moderately thick)	1.00^b^	[0.06, 17.41]
		4 (Extremely thick)	1.07	[0.13, 8.67]
Pharyngeal area at maximum constriction	Larger (i.e., poorer constriction)	0 (Thin)	2.50	[0.39, 16.05]
		1 (Slightly thick)	1.08	[0.22, 5.33]
		2 (Mildly thick)	1.08	[0.18, 6.32]
		3 (Moderately thick)	6.67	[0.69, 64.77]
		4 (Extremely thick)	3.13	[0.51, 19.04]
Vallecular residue	Larger	0 (Thin)	1.00^b^	[0.06, 17.41]
		1 (Slightly thick)	1.07	[0.06, 18.62]
		2 (Mildly thick)	1.00	[0.06, 17.41]
		3 (Moderately thick)	1.00^b^	[0.06, 17.41]
		4 (Extremely thick)	2.13^b^	[0.18, 26.03]
Pyriform sinus residue	Larger	0 (Thin)	0.54	[0.04, 6.58]
		1 (Slightly thick)	1.15	[0.14, 9.38]
		2 (Mildly thick)	2.13^b^	[0.18, 26.03]
		3 (Moderately thick)	0.47^b^	[0.04, 5.72]
		4 (Extremely thick)	0.47^b^	[0.04, 5.72]

*Note.* IDDSI = International Dysphagia Diet Standardisation Initiative; PD = Parkinson disease; CI = confidence interval; UES = upper esophageal sphincter; LVC = laryngeal vestibule closure.

a Extreme = either below the 5th percentile or above the 95th percentile values from the healthy reference distribution.

b Estimated using Haldane–Anscombe correction.

Recognizing that the expected frequencies of extreme values (i.e., ≤ 5%) would translate to less than one person in a cohort of 17 people (i.e., 0.85%) and that a prevalence of 1/17 people would be mathematically represented as a frequency of 6%, we adjusted the threshold for flagging the frequency of extreme values as a finding of interest to a prevalence of at least 2/17participants (12%) in either cohort. A slightly elevated extreme value frequency of 12% was seen on thin liquids for several parameters in the PD cohort: small sip volumes; PAS scores of 2 or worse; prolonged timing measures for SRT and short UES opening duration; and narrow UES opening diameter. On other consistencies, a 12% frequency of extreme values was seen in the PD cohort for large sip volume (slightly and mildly thick), prolonged SRT (slightly thick), a prolonged HYB-to-UESO interval (mildly thick), prolonged UES opening duration (slightly and moderately thick), shortened time-to-LVC (slightly thick) and prolonged time-to-LVC (mildly and moderately thick), short LVC duration (moderately thick), wide UES opening diameter (extremely thick), and for measures of pyriform sinus residue on slightly thick liquids. In the healthy control group, 12% frequencies of extreme values were found for large sip volume (thin and slightly thick), short SRT (slightly thick), a short HYB-to-UESO interval (slightly thick) but a prolonged HYB-to-UES-opening interval on mildly thick liquids, long UES opening (thin and mildly thick), short time-to-LVC (thin), prolonged time-to-LVC (moderately thick), prolonged LVC duration (slightly and mildly thick), wider UES opening diameter (extremely thick), poor pharyngeal constriction (thin and extremely thick), and pyriform sinus residue (thin and slightly thick liquids). Markedly increased frequencies of extreme values, > 12%, were seen in the PwPD as follows:

a) prolonged SRT on mildly and moderately thick liquids;
b) prolonged UES opening duration on thin liquids;
c) prolonged duration of LVC on all consistencies; and
d) poor pharyngeal constriction on all consistencies.

In the healthy control group, extreme value frequencies > 12% were seen for a prolonged HYB-UESO interval on moderately thick liquids, prolonged LVC duration on thin liquids, and poor pharyngeal constriction on slightly and mildly thick liquids. Importantly, however, the ORs failed to show any significant cohort differences in the frequencies of extreme values for any parameter.

## Discussion

### Summary of Findings

This study identifies the participant-level prevalence of atypical and extreme values for several swallowing parameters in a cohort of people with mild PD and a group of age- and sex-matched healthy controls. Elevated frequencies (i.e., > 25%) of atypical values on the majority of consistencies were noted in both cohorts for large sip volumes, prolonged SRTs, increased pharyngeal area at maximum constriction, and vallecular and pyriform residue. Atypical values that were limited to specific consistencies (but common to both cohorts) were also seen for penetration–aspiration on thin liquids, prolonged time-to-LVC on extremely thick liquids, prolonged LVC duration on thin and slightly thick liquids, prolonged UES opening duration on thin and slightly thick liquids, and larger UES opening diameter on slightly and extremely thick liquids. These results are in keeping with our hypotheses. Considering that frequencies > 25% for these atypical values were seen in both groups, these findings represent alterations in swallowing that can be expected with normal aging, independent of any modulating influence from a diagnosis of PD. Similarly, although elevated frequencies of extreme values were seen for several parameters in both cohorts, ORs failed to show any significant cohort differences in extreme value frequencies for any parameter.

By contrast, certain parameters in these data showed increased frequencies of atypical values in only one cohort, suggesting that the presence of PD influenced the presence or absence of the phenomenon. Interestingly, these group differences in the frequencies of atypical values occurred in both directions (i.e., both for values below the 25th percentile, and for values above the 75th percentile of the healthy reference distribution). These differences in frequency might be interpreted to reflect either the increased presence of risk or, conversely, reduced evidence of a possible compensation. For example, atypical wide UES opening diameter was seen in > 25% of the healthy control group on all consistencies, suggesting a possible compensation in healthy older adults to facilitate better bolus clearance. This phenomenon was also seen in the PD cohort on slightly and extremely thick liquids, but was missing on liquids in the mildly thick to moderately thick range, while the opposite pattern, of unusually narrow UES opening diameter, was seen in the PD cohort with thin and slightly thick liquids. This could suggest a role for increased bolus thickness in enhancing sensory input. Other parameters, for which there was a > 25% frequency of atypical values only in the healthy cohort, were penetration–aspiration on slightly thick liquids and a prolonged hyoid-burst-to-UES-opening interval on moderately thick liquids.

Increased frequencies of atypical values that were unique to the PD cohort included a multiple-swallows-per-bolus pattern on thin liquids; a short HYB-to-UES opening interval on extremely thick liquids; prolonged UES opening duration on mildly, moderately, and extremely thick liquids; prolonged time-to-LVC on thin, slightly, mildly, and moderately thick consistencies; prolonged LVC duration on mildly, moderately, and extremely thick liquids; and narrow UES diameter on thin and slightly thick liquids. Notably, however, when ORs were used to compare the frequencies of atypical values between PwPD and the control group, only a few parameters showed significant differences. These were findings of significantly higher frequencies in the PwPD of (a) a short HYB-to-UESO interval on extremely thick liquids, (b) prolonged UES opening duration on slightly and moderately thick liquids, (c) prolonged time-to-LVC on slightly and mildly thick liquids, (d) prolonged LVC duration on slightly and mildly thick liquids, and (e) narrow UES diameter on thin liquids. For all other comparisons, the lower confidence interval boundary for the ORs fell below a value of 1.0, suggesting that the comparisons were underpowered. Caution should, therefore, be exercised when considering the possible implications of these trends.

### Findings in Context

Our findings expand upon the results of a previous study by Ellerston et al. (Ellerston et al., 2016), which used a similar approach of exploring the frequencies of abnormal measures of swallowing in 34 PwPD. Ellerston et al. (2016) identified delayed airway protection and reduced pharyngeal constriction as the most common pharyngeal swallowing abnormalities in their participants and described reduced hyoid elevation, prolonged pharyngeal transit time, and abnormalities of UES opening as occurring rarely or never in their PD cohort. To some degree, our results support these observations. Our participants with PD did show > 25% frequencies of long SRT, prolonged time-to-LVC, and poor pharyngeal constriction. Similarly, they did not show > 25% frequencies of atypical timing for the HYB to UES opening interval (except with extremely thick liquids) or of atypical UES diameter (except with slightly and extremely thick liquids). They did, however, show elevated frequencies of prolonged LVC duration and prolonged UES opening duration, which were not noted in the Ellerston et al. (2016) study. An important observation here is that prolongations of LVC duration and UES opening duration are opposite in direction to changes that would usually be considered to reflect clinical impairment. Discrepancies across the two studies may be explained, in part, by methodologic differences including the selection of measures used for comparison. Ellerston et al. (2016) examined PD participants with controlled volumes of 1, 3, and 20 cc on thin liquids only and did not measure swallowing deficits across the range of thickened liquids. In addition, Ellerston et al. used different criteria to define “normal” and “abnormal” measures compared to the typical versus “atypical” definitions used in our study (K. Kendall, personal communication, September 25, 2020).

Similarly, Baijens et al. (2011) conducted a study of swallowing in 10 PwPD who reported symptoms of dysphagia compared to a group of healthy controls using videofluoroscopy. They found that, despite the presence of “disturbed swallow physiology” in their PD cohort, significant group differences were limited to the finding of delayed velopharyngeal junction closure in the PwPD. The timing of velopharyngeal junction closure was used as an index of the end of the oral phase in the Baijens et al. (2011) study, and the timing of other events, such as LVC, was measured relative to that event. In our study, we used the frame of bolus passing mandible to mark the end of the oral phase, and calculated SRT relative to that event. Differences between the two studies maybe partially explained by poor interrater reliability for several parameters in the Baijens et al. (2011) study, particularly parameters relating to velopharyngeal junction and UES events. In addition, differences in eligibility criteria and videofluoroscopy protocols were noted across the two studies with trials limited to three 10-cc trials of thin liquid in the Baijens et al. study.

More recently, Nascimento et al. (2020) characterized oropharyngeal swallowing physiology in 50 PwPD, specifically assessing whether dopaminergic states affect swallow function. They found time-to-LVC and time-to-laryngeal-vestibule-opening (both relative to GPJO) to be significantly longer in PwPD in the ON state compared to a group of healthy young controls. These observations are consistent with the findings of > 25% frequency of prolonged SRT, time-to-LVC, and LVC duration in the PwPD in our study. Nascimento et al. also observed that, although swallowing safety improved with thicker bolus consistencies in their participants with PD, this came with the trade-off of increased residue. Interestingly, we did not observe atypical amounts of residue in > 25% of our PwPD cohort, except with slightly thick liquids. Here, however, differences in the types of thickener and contrast medium used may have contributed to different results, together with different thresholds for considering residue to be of concern. Notably, however, our study also found increased frequencies of prolonged time-to-LVC on extremely thick liquids in the healthy control group as well as vallecular residue on all consistencies in the current study, except for extremely thick liquids. Such evidence of atypical values in healthy older adults brings into question whether atypical findings can be considered characteristic of PD.

### Contextualizing Findings for Specific Swallowing Parameters

We found an increased frequency of atypical narrow UES opening diameters on thin fluids in our PD cohort. This finding is in keeping with previous histochemical analyses showing an increased percentage of atrophied myofibers in pharyngeal muscle tissue from individuals with PD, which were interpreted to be related to increased tone in the UES (Mu et al., 2012). Conversely, a high frequency of atypical wide UES opening diameter was seen in our healthy control cohort on all consistencies. This is discordant with findings published by Shaw et al. (1995), who suggested that the extent of UES opening during passage of the bolus diminishes with age. A study by Kern et al. (1999) reported that the anteroposterior UES diameter was significantly reduced in the elderly for 5-ml barium boluses, but not for 10-ml boluses. Differences across studies may, therefore, be attributable to sip volume. In our study, the instructions were to take a comfortable sip size, resulting in median sip volumes of 12 ml for the thin to mildly thick liquids and 6 ml for the moderately and extremely thick liquids, which were taken by teaspoon.

In addition to mixed findings regarding UES opening diameter, we found significantly increased frequencies of atypical prolonged UES opening duration in the control cohort on thin and slightly thick fluids. This phenomenon was also observed in the participants with PD, with double to 6 times the odds on all consistencies ranging from thin to extremely thick fluids. The values of UES opening duration on thin liquid swallows in the PD cohort in our study are slightly shorter than those reported by Curtis et al. (2020a) for self-administered thin liquid sips in a sample of 40 participants with early to midstage PD. Interestingly, Curtis et al. (2020a) reported a significant association between short UES opening duration and penetration–aspiration in their study participants. Our findings are also in keeping with those published by Nagaya et al. (1998), who reported that patients with PD had significantly longer durations of UES opening than elderly controls, who, in turn, had significantly longer durations of UES opening compared to healthy younger controls. Our data show an overlapping finding of prolonged UES duration on thin and slightly thick consistencies in both the healthy control and PD cohorts, but an isolated finding in the PD cohort on mildly to extremely thick liquids. This suggests that while this can be expected as part of the normal aging process, the phenomenon may be more common in the context of PD. As mentioned previously, it should be noted the direction of this change does not suggest impairment, but, may, instead reflect some sort of compensation, perhaps to facilitate bolus clearance.

Neither the healthy control nor the PD group in our study was found to have any instances of incomplete LVC. This is in keeping with results from Dumican and Watts (2020), who observed complete LVC in a cohort of individuals with nonadvanced PD. We did find increased frequencies of prolonged time-to-LVC across all consistencies in our PD cohort. Cardinal signs of PD including hypokinesia, slowness of movement (bradykinesia), difficulty in initiating movement, and muscular rigidity have been associated with delayed LVC and increased risk of penetration and aspiration in previous studies (Argolo et al., 2015; Ellerston et al., 2016; Gaeckle et al., 2019; Schiffer & Kendall, 2019). Even though our study sample included individuals with mild PD, who had low rates of penetration–aspiration, our findings are concordant with those reported by Ellerston et al. (2016) who identified delayed airway closure as the most common pharyngeal swallowing deficit in a sample with more advanced PD. Another explanation for delayed airway closure timing in PwPD may be reduced pharyngolaryngeal sensation, impacting the timing and effort of airway protective gestures (Mu et al., 2013; Troche et al., 2014).

A > 25% frequency of prolonged LVC duration was also observed in the PwPD on all consistencies and in the healthy older adults on thin and slightly thick fluids. Taken together with the observations of prolonged UES opening, we propose that these atypically long LVC durations may reflect a compensatory mechanism in the PD group.

### Limitations

Our study is not without limitations. First, our small sample only included individuals with mild PD (H & Y I–II); therefore, the generalizability of our findings across different presentations and severities of PD is unclear. Second, all participants with PD were examined in the medication “on” state, limiting our findings to patients who are actively taking medications. Third, discrepancies between our results and other studies may be due to differences in study design, sample size, assessment tools, and assessment time point relative to the onset of the disease. For example, in our study, patients were examined with an uncontrolled volume cup-sip during videofluoroscopy, whereas other studies have used controlled volume single sip trials.

The small sample sizes for both cohorts in our study also represents a limitation with respect to comparing the frequency of atypical values, and especially the frequency of extreme values between cohorts. As noted above, for the majority of parameters, the lower confidence interval boundary for the OR comparisons regarding atypical values fell below a threshold of 1, such that the observed group differences in frequency failed to reach significance. The cohort sample sizes of 17 were sufficient to reveal significantly different odds for atypically long values of UES opening duration and LVC duration. However, in all other cases, the presence of similar trends in the control group made it impossible to confidently discern whether the PwPD were more likely to display unusual values. Indeed, given the frequencies of atypical values seen in the control group, this study was only powered to detect cohort differences in which the frequency of an atypical finding in one group was at least twofold the frequency seen in the other group. In other words, small-to-medium magnitude differences in swallowing measures may have been present between the two groups but undetected due to the small sample size. Post hoc sample size calculations show that even if both cohorts had included 60 participants (for a total study sample size of 120), and assuming a prevalence of 25% for atypical values in the control group, ORs or logistic regression analyses would only be adequately powered to detect at least a 1.7-fold difference in the frequency of atypical values. With respect to detecting differences in the frequency of extreme values, a study with 60 participants per cohort would only be adequately powered to detect fourfold or greater differences. As such, the findings of our study should be considered preliminary.

## Conclusions

Our findings suggest that the individuals in our PD cohort, who had mild disease, and who were studied in medication “on” state, did not show clear evidence of impairments in pharyngeal swallowing physiology. In fact, for all parameters in which a high frequency of atypical values suggestive of impairment were seen in the PwPD, the same finding was observed in the age-matched healthy control group. Findings suggestive of impairment in both groups, relative to reference values for healthy adults under age 60 years, included prolonged SRTs, poor pharyngeal constriction, and pharyngeal residue. In the two cases where significantly higher frequencies of atypical values were seen in the PwPD, these atypical values of prolonged LVC and UES opening duration were in the direction of compensation rather than impairment. This study points to the need for much larger sample sizes in order to confidently differentiate the effects of PD on swallowing from those seen in healthy aging.

## Supplementary Material

10.1044/2021_JSLHR-21-00084SMS1 Supplemental Material S1 This supplement contains descriptive statistics for measures of swallowing based on duplicated blinded review of videofluoroscopies, performed according to the ASPEKT Method.

## References

[bib1] Argolo, N. , Sampaio, M. , Pinho, P. , Melo, A. , & Nóbrega, A. C. (2015). Videofluoroscopic predictors of penetration–aspiration in Parkinson's disease patients. Dysphagia, 30(6), 751–758. https://doi.org/10.1007/s00455-015-9653-y 26492880 10.1007/s00455-015-9653-y

[bib2] Baijens, L. W. J. , & Speyer, R. (2009). Effects of therapy for dysphagia in Parkinson's disease: Systematic review. Dysphagia, 24(1), 91–102. https://doi.org/10.1007/s00455-008-9180-1 18931877 10.1007/s00455-008-9180-1

[bib3] Baijens, L. W. J. , Speyer, R. , Passos, V. L. , Pilz, W. , Roodenburg, N. , & Clave, P. (2011). Swallowing in Parkinson patients versus healthy controls: Reliability of measurements in videofluoroscopy. Gastroenterology Research and Practice, 2011, 380682. https://doi.org/10.1155/2011/380682 21977026 10.1155/2011/380682 PMC3185253

[bib4] Barbon, C. E. A. , Chepeha, D. B. , Hope, A. J. , Peladeau-Pigeon, M. , Waito, A. A. , & Steele, C. M. (2020). Mechanisms of impaired swallowing on thin liquids following radiation treatment for oropharyngeal cancer. Journal of Speech, Language, and Hearing Research, 63(9), 2870–2879. https://doi.org/10.1044/2020_JSLHR-19-00220 10.1044/2020_JSLHR-19-00220 PMC7890220 32755497

[bib5] Beyer, M. K. , Herlofson, K. , Årsland, D. , & Larsen, J. P. (2001). Causes of death in a community-based study of Parkinson's disease. Acta Neurologica Scandinavica, 103(1), 7–11. https://doi.org/10.1034/j.1600-0404.2001.00191.x 11153892 10.1034/j.1600-0404.2001.00191.x

[bib6] Broadfoot, C. K. , Abur, D. , Hoffmeister, J. D. , Stepp, C. E. , & Ciucci, M. R. (2019). Research-based updates in swallowing and communication dysfunction in Parkinson disease: Implications for evaluation and management. Perspectives of the ASHA Special Interest Groups, 4(5), 825–841. https://doi.org/10.1044/2019_PERS-SIG3-2019-0001 32104723 10.1044/2019_pers-sig3-2019-0001 PMC7043100

[bib7] Cereda, E. , Cilia, R. , Klersy, C. , Canesi, M. , Zecchinelli, A. L. , Mariani, C. B. , Tesei, S. , Sacilotto, G. , Meucci, N. , Zini, M. , Isaias, I. U. , Cassani, E. , Goldwurm, S. , Barichella, M. , & Pezzoli, G. (2014). Swallowing disturbances in Parkinson's disease: A multivariate analysis of contributing factors. Parkinsonism & Related Disorders, 20(12), 1382–1387. https://doi.org/10.1016/j.parkreldis.2014.09.031 25456827 10.1016/j.parkreldis.2014.09.031

[bib8] Curtis, J. A. , Molfenter, S. M., & Troche, M. S. (2020a). Predictors of residue and airway invasion in Parkinson's disease. Dysphagia, 35(2), 220–230. https://doi.org/10.1007/s00455-019-10014-z 31028481 10.1007/s00455-019-10014-z PMC8711115

[bib9] Curtis, J. A. , Molfenter, S. M. , & Troche, M. S. (2020b). Pharyngeal area changes in Parkinson's disease and its effect on swallowing safety, efficiency, and kinematics. Dysphagia, 35(2), 389–398. https://doi.org/10.1007/s00455-019-10052-7 31446478 10.1007/s00455-019-10052-7 PMC7513198

[bib10] Dorsey, E. R. , Constantinescu, R. , Thompson, J. P. , Biglan, K. M. , Holloway, R. G. , Kieburtz, K. , Marshall, F. J. , Ravina, B. M. , Schifitto, G. , Siderowf, A. , & Tanner, C. M. (2007). Projected number of people with Parkinson disease in the most populous nations, 2005 through 2030. Neurology, 68(5), 384–386. https://doi.org/10.1212/01.wnl.0000247740.47667.03 17082464 10.1212/01.wnl.0000247740.47667.03

[bib11] Dumican, M. , & Watts, C. (2020). Predicting airway invasion using screening tools and laryngeal kinematics in people with Parkinson's disease: A pilot study. Journal of Parkinson's Disease, 10(3), 1153–1160. https://doi.org/10.3233/JPD-202044 10.3233/JPD-202044 PMC7458512 32538868

[bib12] Ellerston, J. K. , Heller, A. C. , Houtz, D. R. , & Kendall, K. A. (2016). Quantitative measures of swallowing deficits in patients with Parkinson's disease. Annals of Otology, Rhinology & Laryngology, 125(5), 385–392. https://doi.org/10.1177/0003489415617774 10.1177/0003489415617774 26602905

[bib13] Gaeckle, M. , Domahs, F. , Kartmann, A. , Tomandl, B. , & Frank, U. (2019). Predictors of penetration–aspiration in Parkinson's disease patients with dysphagia: A retrospective analysis. Annals of Otology, Rhinology & Laryngology, 128(8), 728–735. https://doi.org/10.1177/0003489419841398 10.1177/0003489419841398 30939890

[bib14] Hoehn, M. M. , & Yahr, M. D. (1967). Parkinsonism: Onset, progression and mortality. Neurology, 17(5), 427–442. 6067254 10.1212/wnl.17.5.427

[bib15] Jardine, M. , Miles, A. , & Allen, J. E. (2018). Swallowing function in advanced age. Current Opinions in Otolaryngology & Head and Neck Surgery, 26(6), 367–374. https://doi.org/10.1097/MOO.0000000000000485 10.1097/MOO.0000000000000485 30234658

[bib16] Jones, C. A. , & Ciucci, M. R. (2016). Multimodal swallowing evaluation with high-resolution manometry reveals subtle swallowing changes in early and mid-stage Parkinson disease. Journal of Parkinson's Disease, 6(1), 197–208. https://doi.org/10.3233/JPD-150687 10.3233/JPD-150687 PMC4816667 26891176

[bib17] Jones, C. A. , Hoffman, M. R. , Lin, L. , Abdelhalim, S. , Jiang, J. J. , & McCulloch, T. M. (2018). Identification of swallowing disorders in early and mid-stage Parkinson's disease using pattern recognition of pharyngeal high-resolution manometry data. Neurogastroenterology & Motility, 30(4), e13236. https://doi.org/10.1111/nmo.13236 29143418 10.1111/nmo.13236 PMC5878743

[bib50] Kahrilas, P. J. , Logemann, J. A. , Lin, S. , & Ergun, G. A. (1992). Pharyngeal clearance during swallowing: A combined manometric and videofluoroscopic study. Gastroenterology, 103(1), 128–136. https://doi.org/10.1016/0016-5085(92)91105-D 1612322 10.1016/0016-5085(92)91105-d

[bib18] Kalf, J. G. , de Swart, B. J. M. , Bloem, B. R. , & Munneke, M. (2012). Prevalence of oropharyngeal dysphagia in Parkinson's disease: A meta-analysis. Parkinsonism & Related Disorders, 18(4), 311–315. https://doi.org/10.1016/j.parkreldis.2011.11.006 22137459 10.1016/j.parkreldis.2011.11.006

[bib19] Kendall, K. A. (2002). Oropharyngeal swallowing variability. The Laryngoscope, 112(3), 547–551. https://doi.org/10.1097/00005537-200203000-00025 12148869 10.1097/00005537-200203000-00025

[bib20] Kendall, K. A. , & Leonard, R. J. (2001). Pharyngeal constriction in elderly dysphagic patients compared with young and elderly nondysphagic controls. Dysphagia, 16(4), 272–278. https://doi.org/10.1007/s00455-001-0086-4 11720403 10.1007/s00455-001-0086-4

[bib21] Kendall, K. A. , McKenzie, S. , Leonard, R. J. , Goncalves, M. I. , & Walker, A. (2000). Timing of events in normal swallowing: A videofluoroscopic study. Dysphagia, 15(2), 74–83. https://doi.org/10.1007/s004550010004 10758189 10.1007/s004550010004

[bib22] Kern, M. , Bardan, E. , Arndorfer, R. , Hofmann, C. , Ren, J. , & Shaker, R. (1999). Comparison of upper esophageal sphincter opening in healthy asymptomatic young and elderly volunteers. Annals of Otology, Rhinology & Laryngology, 108(10), 982–989. https://doi.org/10.1177/000348949910801010 10.1177/000348949910801010 10526854

[bib23] Kim, Y. H. , Oh, B. M. , Jung, I. Y. , Lee, J. C. , Lee, G. J. , & Han, T. R. (2015). Spatiotemporal characteristics of swallowing in Parkinson's disease. The Laryngoscope, 125(2), 389–395. https://doi.org/10.1002/lary.24869 25093527 10.1002/lary.24869

[bib24] Lawson, R. (2004). Small sample confidence intervals for the odds ratio. Communication in Statistics - Simulation and Computation, 33(4), 1095–1113. https://doi.org/10.1081/SAC-200040691

[bib51] Leonard, R. , Kendall, K. A. , & McKenzie, S. (2004). UES opening and cricopharyngeal bar in nondysphagic elderly and nonelderly adults. Dysphagia, 19(3), 182–191. https://doi.org/10.1007/s00455-004-0005-6 15383948 10.1007/s00455-004-0005-6

[bib25] Mancopes, R. , Gandhi, P. , Smaoui, S. , & Steele, C. M. (2021). Which physiological swallowing parameters change with healthy aging. OBM Geriatrics, 5(1), 16. https://doi.org/10.21926/obm.geriatr.2101153 10.21926/obm.geriatr.2101153 PMC8330408 34350402

[bib26] Mancopes, R. , Peladeau-Pigeon, M. , Barrett, E. , Guran, A. , Smaoui, S. , Schmidt-Pasqualoto, A. S. , & Steele, C. M. (2020). Quantitative videofluoroscopic analysis of swallowing physiology and function in individuals with chronic obstructive pulmonary disease (COPD). Journal of Speech, Language, and Hearing Research, 63(11), 3643–3658. https://doi.org/10.1044/2020_JSLHR-20-00154 10.1044/2020_JSLHR-20-00154 PMC8582841 33105085

[bib27] Marras, C. , Beck, J. C. , Bower, J. H. , Roberts, E. , Ritz, B. , Ross, G. W. , Abbott, R. D. , Savica, R. , Van Den Eeden, S. K. , Willis, A. W. , Tanner, C. M. , & Parkinson's Foundation P4 Group. (2018). Prevalence of Parkinson's disease across North America. NPJ Parkinson's Disease, 4, 21. https://doi.org/10.1038/s41531-018-0058-0 10.1038/s41531-018-0058-0 PMC6039505 30003140

[bib28] Miller, N. , Allcock, L. , Hildreth, A. J. , Jones, D. , Noble, E. , & Burn, D. J. (2009). Swallowing problems in Parkinson disease: frequency and clinical correlates. Journal of Neurology, Neurosurgery & Psychiatry, 80(9), 1047–1049. https://doi.org/10.1136/jnnp.2008.157701 10.1136/jnnp.2008.157701 19028764

[bib29] Molfenter, S. M. , Lenell, C. , & Lazarus, C. L. (2019). Volumetric changes to the pharynx in healthy aging: Consequence for pharyngeal swallow mechanics and function. Dysphagia, 34(1), 129–137. https://doi.org/10.1007/s00455-018-9924-5 30039259 10.1007/s00455-018-9924-5 PMC6344328

[bib30] Molfenter, S. M. , & Steele, C. M. (2014). Use of an anatomical scalar to control for sex-based size differences in measures of hyoid excursion during swallowing. Journal of Speech, Language, and Hearing Research, 57(3), 768–778. https://doi.org/10.1044/2014_JSLHR-S-13-0152 10.1044/2014_JSLHR-S-13-0152 PMC4318235 24686851

[bib31] Mu, L. , Sobotka, S. , Chen, J. , Su, H. , Sanders, I. , Adler, C. H. , Shill, H. A. , Caviness, J. N. , Samanta, J. E. , Beach, T. G. , & Arizona Parkinson's Disease Consortium. (2012). Altered pharyngeal muscles in Parkinson disease. Journal of Neuropathology & Experimental Neurology, 71(6), 520–530. https://doi.org/10.1097/NEN.0b013e318258381b 22588389 10.1097/NEN.0b013e318258381b PMC3358551

[bib32] Mu, L. , Sobotka, S. , Chen, J. , Su, H. , Sanders, I. , Nyirenda, T. , Adler, C. H. , Shill, H. A. , Caviness, J. N. , Samanta, J. E. , Sue, L. I. , Beach, T. G. , & Arizona Parkinson's Disease Consortium. (2013). Parkinson disease affects peripheral sensory nerves in the pharynx. Journal of Neuropathology & Experimental Neurology, 72(7), 614–623. https://doi.org/10.1097/NEN.0b013e3182965886 23771215 10.1097/NEN.0b013e3182965886 PMC3695629

[bib33] Nagaya, M. , Kachi, T. , Yamada, T. , & Igata, A. (1998). Videofluorographic study of swallowing in Parkinson's disease. Dysphagia, 13(2), 95–100. https://doi.org/10.1007/PL00009562 9513304 10.1007/PL00009562

[bib34] Namasivayam-MacDonald, A. M. , Barbon, C. E. A. , & Steele, C. M. (2018). A review of swallow timing in the elderly. Physiology & Behavior, 184(1), 12–26. https://doi.org/10.1016/j.physbeh.2017.10.023 29101012 10.1016/j.physbeh.2017.10.023 PMC5742298

[bib35] Nascimento, W. V. , Arreola, V. , Sanz, P. , Necati, E. , Bolivar-Prados, M. , Michou, E. , Ortega, O. , & Clavé, P. (2020). Pathophysiology of swallowing dysfunction in Parkinson disease and lack of dopaminergic impact on the swallow function and on the effect of thickening agents. Brain Sciences, 10(9), 609. https://doi.org/10.3390/brainsci10090609 10.3390/brainsci10090609 PMC7563552 32899758

[bib36] Plowman-Prine, E. K. , Sapienza, C. M. , Okun, M. S. , Pollock, S. L. , Jacobson, C. , Wu, S. S. , & Rosenbek, J. C. (2009). The relationship between quality of life and swallowing in Parkinson's disease. Movement Disorders, 24(9), 1352–1358. https://doi.org/10.1002/mds.22617 19425089 10.1002/mds.22617 PMC3614344

[bib37] Pringsheim, T. , Jette, N. , Frolkis, A. , & Steeves, T. D. (2014). The prevalence of Parkinson's disease: A systematic review and meta-analysis. Movement Disorders, 29(13), 1583–1590. https://doi.org/10.1002/mds.25945 24976103 10.1002/mds.25945

[bib38] Robbins, J. A. , Hamilton, J. W. , Lof, G. L. , & Kempster, G. B. (1992). Oropharyngeal swallowing in normal adults of different ages. Gastroenterology, 103(3), 823–829. https://doi.org/10.1016/0016-5085(92)90013-O 1499933 10.1016/0016-5085(92)90013-o

[bib39] Robbins, J. A. , Logemann, J. A. , & Kirshner, H. S. (1986). Swallowing and speech production in Parkinson's disease. Annals of Neurology, 19(3), 283–287. https://doi.org/10.1002/ana.410190310 3963773 10.1002/ana.410190310

[bib40] Rosenbek, J. C. , Robbins, J. A. , Roecker, E. B. , Coyle, J. L. , & Wood, J. L. (1996). A penetration–aspiration scale. Dysphagia, 11(2), 93–98. https://doi.org/10.1007/BF00417897 8721066 10.1007/BF00417897

[bib41] Schiffer, B. L. , & Kendall, K. (2019). Changes in timing of swallow events in Parkinson's disease. Annals of Otology, Rhinology & Laryngology, 128(1), 22–27. https://doi.org/10.1177/0003489418806918 10.1177/0003489418806918 30328706

[bib42] Shaw, D. W. , Cook, I. J. , Gabb, M. , Holloway, R. H. , Simula, M. E. , Panagopoulos, V. , & Dent, J. (1995). Influence of normal aging on oral-pharyngeal and upper esophageal sphincter function during swallowing. American Journal of Physiology, 268(3), G389–G396. https://doi.org/10.1152/ajpgi.1995.268.3.G389 10.1152/ajpgi.1995.268.3.G389 7900799

[bib43] Steele, C. M. , Peladeau-Pigeon, M. , Barbon, C. A. E. , Guida, B. T. , Namasivayam-MacDonald, A. M. , Nascimento, W. V. , Smaoui, S. , Tapson, M. S. , Valenzano, T. J. , Waito, A. A. , & Wolkin, T. S. (2019). Reference values for healthy swallowing across the Range From thin to extremely thick liquids. Journal of Speech, Language, and Hearing Research, 62(5), 1338–1363. https://doi.org/10.1044/2019_JSLHR-S-18-0448 10.1044/2019_JSLHR-S-18-0448 PMC6808317 31021676

[bib44] Steele Swallowing Lab. (2020). ASPEKT Method: Supplemental material. https://steeleswallowinglab.ca/srrl/wp-content/uploads/ASPEKT-Method-Reference-Value-Tables-V1.3.pdf

[bib45] Stokely, S. L. , Peladeau-Pigeon, M. , Leigh, C. , Molfenter, S. M. , & Steele, C. M. (2015). The relationship between pharyngeal constriction and post-swallow residue. Dysphagia, 30(3), 349–356. https://doi.org/10.1007/s00455-015-9606-5 25920993 10.1007/s00455-015-9606-5 PMC4469308

[bib46] Suttrup, I. , & Warnecke, T. (2016). Dysphagia in Parkinson's disease. Dysphagia, 31(1), 24–32. https://doi.org/10.1007/s00455-015-9671-9 26590572 10.1007/s00455-015-9671-9

[bib47] Troche, M. S. , Brandimore, A. E. , Okun, M. S. , Davenport, P. W. , & Hegland, K. W. (2014). Decreased cough sensitivity and aspiration in Parkinson disease. Chest, 146(5), 1294–1299. https://doi.org/10.1378/chest.14-0066 24968148 10.1378/chest.14-0066 PMC4219343

[bib48] Waito, A. A. , Plowman, E. K. , Barbon, C. E. A. , Peladeau-Pigeon, M. , Tabor-Gray, L. , Magennis, K. , Robison, R. , & Steele, C. M. (2020). A cross-sectional, quantitative videofluoroscopic analysis of swallowing physiology and function in individuals with amyotrophic lateral sclerosis. Journal of Speech, Language, and Hearing Research, 63(4), 948–962. https://doi.org/10.1044/2020_JSLHR-19-00051 10.1044/2020_JSLHR-19-00051 PMC7242989 32310713

[bib49] Waito, A. A. , Tabor-Gray, L. , Steele, C. M. , & Plowman, E. K. (2018). Reduced pharyngeal constriction is associated with impaired swallowing efficiency in amyotrophic lateral sclerosis (ALS). Neurogastroenterology and Motility, 30(12), e13450. https://doi.org/10.1111/nmo.13450 30129164 10.1111/nmo.13450 PMC6249041

