# Supplementary material for: The Frequency of Atypical and Extreme Values for Pharyngeal Phase Swallowing Measures in Mild Parkinson Disease Compared to Healthy Aging
Source: J Speech Lang Hear Res. 2021 Jul 27;64(8):3032–50. doi: 10.1044/2021_JSLHR-21-00084 (PMC8740655; doi:10.1044/2021_JSLHR-21-00084)
Supplement: Supplemental Material S1 [file JSLHR-64-3032-s001.pdf]

Supplementary Tables

This supplement contains descriptive statistics for measures of swallowing based on duplicated blinded review of videofluoroscopies, performed according to the ASPEKT Method (Steele et al., 2019). For continuous parameters, means and standard deviations are reported, as well as percentile values. For categorical parameters, values for percentiles are reported.

The parameters of interest are as follows:

- sip volume, milliliters;
- the number of swallows per bolus;
- the Penetration-Aspiration Scale (Rosenbek et al., 1996) score seen on the initial swallow of each bolus;
- the worst (i.e. maximum) Penetration-Aspiration Scale score seen across all swallows for each bolus;
- laryngeal vestibule closure (LVC) integrity (complete, partial or incomplete);
- swallow reaction time (defined as the interval between bolus passing mandible and onset of the hyoid burst), in milliseconds;
- the hyoid-burst-onset-to-upper-esophageal-sphincter-opening interval, in milliseconds;
- upper esophageal sphincter (UES) opening duration, in milliseconds;
- time-to-LVC, in milliseconds, defined as the interval between onset of the hyoid burst and the first frame of most-complete LVC;
- LVC duration, in milliseconds;
- vallecular residue area, pyriform sinus residue area, other pharyngeal residue area and total pharyngeal residue (calculated as the sum of vallecular, pyriform sinus and other pharyngeal residue area), measured in anatomically-scaled units, i.e. $\%(C2\text{-}4)^2$ units;
- pharyngeal area on the frame of maximum pharyngeal constriction ("PhAMPC"), in $\%(C2\text{-}4)^2$ units; and
- UES diameter at maximum distension, in %(C2-4) units.

It should be noted that measures of pharyngeal area at rest, bolus location at swallow onset, and hyoid kinematics are included in the ASPEKT method but are not included in this manuscript. Supplementary Table 1 contains data for the original reference sample of 38 healthy adults aged under 60 years of age described by Steele and colleagues (Steele et al., 2019). The data in these tables expand upon the descriptive statistics of means and standard deviations provided in the original article. Additional information regarding the ASPEKT method can be found in the original article (Steele et al., 2019).

In the current study, these healthy reference values were used to classify data from two new cohorts (a cohort of healthy older adults and a cohort of adults with Parkinson Disease), using the following classifications:

- Abnormal (low): Below the 5th percentile of the healthy reference range;
- Atypical (low): Below the 25th percentile of the healthy reference range;
- Typical: Within the healthy reference interquartile range, i.e., between the 25th and 75th percentiles;
- Atypical (high): Above the 75th percentile of the healthy reference range;

- Abnormal (high): Above the 95th percentile of the healthy reference range.

Supplementary Tables 2 and 3 contain descriptive statistics for healthy older cohort and the participants with PD, respectively.

References:

Rosenbek, J. C., Robbins, J. A., Roecker, E. B., Coyle, J. L., & Wood, J. L. (1996). A penetration–aspiration scale. *Dysphagia, 11*(2), 93–98.

Steele, C. M., et al. (2019). Reference values for healthy swallowing across the range from thin to extremely thick liquids. *Journal of Speech, Language, and Hearing Research, 62*(5), 1338-1363. https://doi.org/10.1044/2019_JSLHR-S-18-0448

Supplementary Table 1. Descriptive statistics for the healthy reference sample aged < 60 years of age, studied by Steele et al. (2019).

**Parameter**	**Unit**	**Consistency**	**Mean**	**Standard Deviation**	**p5**	**p25**	**p50**	**p75**	**p95**
Sip Volume	ml	Thin	11	5	4	8	11	15	20
		Slightly thick	9	4	3	6	9	12	16
		Mildly thick	9	4	3	6	8	11	18
		Moderately thick	5	3	2	3	4	6	11
		Extremely thick	5	3	2	3	5	6	10
Number of Swallows per Bolus	Number	Thin	1	1	1	1	1	1	2
		Slightly thick	1	0	1	1	1	1	2
		Mildly thick	1	1	1	1	1	1	2
		Moderately thick	1	0	1	1	1	1	2
		Extremely thick	1	1	1	1	1	1	2

Penetration-Aspiration Scale Score	Categorical Score 1-8	Thin	N/A	N/A	1	1	1	1	2
		Slightly thick	N/A	N/A	1	1	1	1	2
		Mildly thick	N/A	N/A	1	1	1	1	1
		Moderately thick	N/A	N/A	1	1	1	1	1
		Extremely thick	N/A	N/A	1	1	1	1	1
Worst Penetration-Aspiration Scale Score	Categorical Score 1-8	Thin	N/A	N/A	1	1	1	1	2
		Slightly thick	N/A	N/A	1	1	1	1	2
		Mildly thick	N/A	N/A	1	1	1	1	2
		Moderately thick	N/A	N/A	1	1	1	1	2
		Extremely thick	N/A	N/A	1	1	1	1	1
LVC Integrity	Complete/ Partial/ Incomplete	Thin	N/A	N/A	Complete	Complete	Complete	Complete	Complete
		Slightly thick	N/A	N/A	Complete	Complete	Complete	Complete	Complete
		Mildly thick	N/A	N/A	Complete	Complete	Complete	Complete	Complete
		Moderately thick	N/A	N/A	Complete	Complete	Complete	Complete	Complete
		Extremely thick	N/A	N/A	Complete	Complete	Complete	Complete	Complete

Swallow Reaction Time	ms	Thin	130	183	-67	0	67	167	577
		Slightly thick	179	260	-33	0	67	267	797
		Mildly thick	205	286	-33	0	100	300	914
		Moderately thick	315	420	-67	0	100	567	1208
		Extremely thick	364	509	-67	0	67	617	1741
Hyoid-Burst-to-UES-Opening Interval(ms)	ms	Thin	109	57	33	67	100	133	234
		Slightly thick	120	60	23	100	133	167	234
		Mildly thick	124	59	33	92	133	167	209
		Moderately thick	150	56	67	100	133	200	234
		Extremely thick	154	52	67	133	133	200	249
UES Opening Duration	ms	Thin	456	62	367	400	467	500	567
		Slightly thick	441	65	334	400	434	467	567
		Mildly thick	446	80	334	400	434	500	601
		Moderately thick	415	74	300	367	400	467	544
		Extremely thick	404	69	300	367	400	434	547

Time-to-LVC	ms	Thin	185	103	33	100	167	259	399
		Slightly thick	178	89	67	100	167	234	367
		Mildly thick	159	77	33	100	133	200	314
		Moderately thick	154	63	67	100	133	200	267
		Extremely thick	147	57	67	100	133	167	267
LVC Duration	ms	Thin	460	162	300	367	434	500	691
		Slightly thick	446	172	267	367	417	500	701
		Mildly thick	453	134	300	367	434	509	667
		Moderately thick	448	148	300	367	434	500	676
		Extremely thick	434	87	322	367	434	500	601
Vallecular Residue	$\%(C2\text{-}4)^2$	Thin	0%	1%	0%	0%	0%	0%	2%
		Slightly thick	1%	1%	0%	0%	0%	1%	2%
		Mildly thick	1%	1%	0%	0%	0%	1%	3%
		Moderately thick	0%	1%	0%	0%	0%	0%	2%
		Extremely thick	0%	1%	0%	0%	0%	0%	2%

Pyriform Sinus Residue	$\%(C2\text{-}4)^2$	Thin	0%	1%	0%	0%	0%	0%	1%
		Slightly thick	0%	1%	0%	0%	0%	0%	2%
		Mildly thick	0%	1%	0%	0%	0%	0%	2%
		Moderately thick	0%	0%	0%	0%	0%	0%	1%
		Extremely thick	0%	0%	0%	0%	0%	0%	1%
Other Pharyngeal Residue	$\%(C2\text{-}4)^2$	Thin	0%	0%	0%	0%	0%	0%	1%
		Slightly thick	0%	0%	0%	0%	0%	0%	1%
		Mildly thick	0%	0%	0%	0%	0%	0%	1%
		Moderately thick	0%	0%	0%	0%	0%	0%	1%
		Extremely thick	0%	0%	0%	0%	0%	0%	1%
Total Pharyngeal Residue	$\%(C2\text{-}4)^2$	Thin	1%	2%	0%	0%	0%	1%	3%
		Slightly thick	1%	2%	0%	0%	0%	1%	6%
		Mildly thick	1%	2%	0%	0%	1%	2%	5%
		Moderately thick	1%	1%	0%	0%	0%	1%	2%
		Extremely thick	1%	1%	0%	0%	0%	1%	3%

Pharyngeal Area at Maximum Constriction	$\%(C2\text{-}4)^2$	Thin	1%	1%	0%	0%	0%	2%	4%
		Slightly thick	1%	1%	0%	0%	0%	2%	4%
		Mildly thick	2%	2%	0%	0%	1%	3%	6%
		Moderately thick	1%	1%	0%	0%	0%	1%	3%
		Extremely thick	1%	1%	0%	0%	0%	1%	3%
UES Diameter	%(C2-4)	Thin	21%	7%	10%	16%	20%	25%	32%
		Slightly thick	19%	6%	10%	15%	18%	22%	29%
		Mildly thick	18%	5%	11%	14%	18%	22%	27%
		Moderately thick	16%	5%	9%	12%	14%	19%	26%
		Extremely thick	17%	5%	9%	13%	17%	20%	25%

p5 = 5th percentile (p25, p50, p75, p95 = 25th, 50th, 75th, 95th percentiles); ml = milliliters; ms = milliseconds; LVC = Laryngeal Vestibule Closure; UES = Upper Esophageal Sphincter; $\%(C2\text{-}4)^2$ = percent of the squared length of the C2-4 cervical spine; %(C2-4) = percent of the length of the C2-4 cervical spine; N/A = not available.

Supplementary Table 2. Descriptive statistics for videofluoroscopic measures of swallowing in healthy participants aged 53-82.

**Parameter**	**Unit**	**Consistency**	**Mean**	**Standard Deviation**	**p5**	**p25**	**p50**	**p75**	**p95**
Sip Volume	ml	Thin	16	8	6	10	16	19	40
		Slightly thick	13	5	6	8	11	17	23
		Mildly thick	12	5	5	9	10	17	21
		Moderately thick	5	2	2	3	6	7	10
		Extremely thick	6	3	2	4	6	8	11
Number of Swallows per Bolus	Number	Thin	1	0	1	1	1	1	2
		Slightly thick	1	1	1	1	1	1	3
		Mildly thick	1	1	1	1	1	1	3
		Moderately thick	1	0	1	1	1	1	1
		Extremely thick	1	0	1	1	1	1	3

Penetration-Aspiration Scale Score	Categorical Score 1-8	Thin	N/A	N/A	1	1	1	1	2
		Slightly thick	N/A	N/A	1	1	1	1	2
		Mildly thick	N/A	N/A	1	1	1	1	2
		Moderately thick	N/A	N/A	1	1	1	1	1
		Extremely thick	N/A	N/A	1	1	1	1	1
Worst Penetration-Aspiration Scale Score	Categorical Score 1-8	Thin	N/A	N/A	1	1	1	1	2
		Slightly thick	N/A	N/A	1	1	1	1	2
		Mildly thick	N/A	N/A	1	1	1	1	2
		Moderately thick	N/A	N/A	1	1	1	1	1
		Extremely thick	N/A	N/A	1	1	1	1	1
LVC Integrity	Complete/Partial/ Incomplete	Thin	N/A	N/A	Complete	Complete	Complete	Complete	Complete
		Slightly thick	N/A	N/A	Complete	Complete	Complete	Complete	Complete
		Mildly thick	N/A	N/A	Complete	Complete	Complete	Complete	Complete
		Moderately thick	N/A	N/A	Complete	Complete	Complete	Complete	Complete
		Extremely thick	N/A	N/A	Complete	Complete	Complete	Complete	Complete

Swallow Reaction Time	ms	Thin	270	315	-70	83	200	267	1108
		Slightly thick	247	300	-28	25	183	275	952
		Mildly thick	327	369	-62	100	200	500	1378
		Moderately thick	451	356	-33	133	400	701	967
		Extremely thick	641	506	-70	200	634	984	1655
Hyoid-Burst-to-UES-Opening Interval(ms)	ms	Thin	102	61	-37	67	100	133	200
		Slightly thick	115	76	-33	67	133	167	234
		Mildly thick	134	82	0	100	133	167	262
		Moderately thick	193	66	67	167	167	234	300
		Extremely thick	191	57	97	150	200	234	304
UES Opening Duration	ms	Thin	488	95	300	434	467	534	671
		Slightly thick	470	89	339	400	450	534	634
		Mildly thick	470	95	367	400	434	500	701
		Moderately thick	407	77	267	367	400	467	567
		Extremely thick	399	76	267	367	400	434	601

Time-to-LVC	ms	Thin	151	131	-50	67	133	200	470
		Slightly thick	153	96	38	100	133	200	437
		Mildly thick	167	131	33	100	133	200	457
		Moderately thick	194	78	100	133	167	234	400
		Extremely thick	180	61	67	133	200	234	270
LVC Duration	ms	Thin	542	224	254	434	500	601	1051
		Slightly thick	510	174	305	400	467	542	929
		Mildly thick	496	166	300	400	467	509	867
		Moderately thick	453	112	334	367	434	500	734
		Extremely thick	462	138	300	400	434	500	874
Vallecular Residue	$\%(C2\text{-}4)^2$	Thin	1%	1%	0%	0%	0%	1%	3%
		Slightly thick	1%	1%	0%	0%	1%	1%	5%
		Mildly thick	1%	1%	0%	0%	0%	1%	3%
		Moderately thick	0%	1%	0%	0%	0%	1%	3%
		Extremely thick	0%	0%	0%	0%	0%	0%	2%

Pyriform Sinus Residue	$\%(C2\text{-}4)^2$	Thin	1%	1%	0%	0%	0%	1%	4%
		Slightly thick	1%	1%	0%	0%	0%	1%	4%
		Mildly thick	1%	1%	0%	0%	0%	1%	3%
		Moderately thick	0%	1%	0%	0%	0%	0%	2%
		Extremely thick	0%	1%	0%	0%	0%	0%	2%
Other Pharyngeal Residue	$\%(C2\text{-}4)^2$	Thin	0%	0%	0%	0%	0%	0%	1%
		Slightly thick	0%	0%	0%	0%	0%	0%	1%
		Mildly thick	0%	1%	0%	0%	0%	0%	2%
		Moderately thick	0%	0%	0%	0%	0%	0%	1%
		Extremely thick	0%	1%	0%	0%	0%	0%	2%
Total Pharyngeal Residue	$\%(C2\text{-}4)^2$	Thin	1%	2%	0%	0%	1%	2%	7%
		Slightly thick	2%	3%	0%	0%	1%	2%	8%
		Mildly thick	2%	2%	0%	0%	1%	3%	6%
		Moderately thick	1%	1%	0%	0%	0%	1%	5%
		Extremely thick	1%	1%	0%	0%	0%	1%	5%

Pharyngeal Area at Maximum Constriction	$\%(C2\text{-}4)^2$	Thin	3%	3%	0%	0%	2%	3%	9%
		Slightly thick	3%	2%	0%	1%	2%	3%	8%
		Mildly thick	4%	5%	0%	1%	2%	4%	11%
		Moderately thick	2%	3%	0%	0%	1%	2%	7%
		Extremely thick	1%	2%	0%	0%	0%	3%	6%
UES Diameter	%(C2-4)	Thin	26%	8%	14%	20%	25%	28%	47%
		Slightly thick	24%	8%	14%	18%	21%	26%	46%
		Mildly thick	24%	8%	12%	18%	22%	28%	44%
		Moderately thick	19%	4%	13%	15%	18%	21%	28%
		Extremely thick	20%	6%	14%	16%	19%	24%	32%

p5 = 5th percentile (p25, p50, p75, p95 = 25th, 50th, 75th, 95th percentiles); ml = milliliters; ms = milliseconds; LVC = Laryngeal Vestibule Closure; UES = Upper Esophageal Sphincter; $\%(C2\text{-}4)^2$ = percent of the squared length of the C2-4 cervical spine; %(C2-4) = percent of the length of the C2-4 cervical spine; N/A = not available.

Supplementary Table 3. Descriptive statistics for videofluoroscopic measures of swallowing in participants with Parkinson Disease.

Parameter	Unit	Consistency	Mean	Standard Deviation	p25	p50	p75
Sip Volume	ml	Thin	11	8	6	11	15
		Slightly thick	11	8	6	12	16
		Mildly thick	12	9	8	13	15
		Moderately thick	5	5	4	6	7
		Extremely thick	7	5	5	6	9
Number of Swallows per Bolus	Number	Thin	1	1	1	1	2
		Slightly thick	1	1	1	1	1
		Mildly thick	1	1	1	1	1
		Moderately thick	1	1	1	1	1
		Extremely thick	1	1	1	1	1
Penetration-Aspiration Scale Score	Categorical Score 1-8	Thin	N/A	N/A	1	1	2
		Slightly thick	N/A	N/A	1	1	2
		Mildly thick	N/A	N/A	1	1	1
		Moderately thick	N/A	N/A	1	1	1
		Extremely thick	N/A	N/A	1	1	1

Worst Penetration-Aspiration Scale Score	Categorical Score 1-8	Thin	N/A	N/A	1	1	2
		Slightly thick	N/A	N/A	1	1	2
		Mildly thick	N/A	N/A	1	1	1
		Moderately thick	N/A	N/A	1	1	1
		Extremely thick	N/A	N/A	1	1	1
LVC Integrity	Complete/Partial/ Incomplete	Thin	N/A	N/A	Complete	Complete	Complete
		Slightly thick	N/A	N/A	Complete	Complete	Complete
		Mildly thick	N/A	N/A	Complete	Complete	Complete
		Moderately thick	N/A	N/A	Complete	Complete	Complete
		Extremely thick	N/A	N/A	Complete	Complete	Complete
Swallow Reaction Time	ms	Thin	278	182	100	256	423
		Slightly thick	253	123	75	178	384
		Mildly thick	393	123	56	133	589
		Moderately thick	512	212	89	295	879
		Extremely thick	508	258	56	322	934
Hyoid-Burst-to-UES-Opening Interval(ms)	ms	Thin	110	88	89	111	145
		Slightly thick	126	93	92	128	181
		Mildly thick	126	99	100	122	156
		Moderately thick	144	116	104	128	183
		Extremely thick	147	115	111	122	200

UES Opening Duration	ms	Thin	533	505	489	523	578
		Slightly thick	506	469	475	500	559
		Mildly thick	485	442	434	478	567
		Moderately thick	466	420	434	484	523
		Extremely thick	444	401	378	445	523
Time-to-LVC	ms	Thin	181	85	100	245	289
		Slightly thick	151	-14	117	211	295
		Mildly thick	136	16	100	211	245
		Moderately thick	189	152	122	200	245
		Extremely thick	180	149	133	200	211
LVC Duration	ms	Thin	574	469	489	523	689
		Slightly thick	573	410	487	545	623
		Mildly thick	564	440	445	534	601
		Moderately thick	499	415	406	506	653
		Extremely thick	496	409	400	478	589
Vallecular Residue	$\%(C2\text{-}4)^2$	Thin	0%	0%	0%	0%	1%
		Slightly thick	0%	0%	0%	0%	1%
		Mildly thick	1%	0%	0%	0%	1%
		Moderately thick	0%	0%	0%	0%	0%
		Extremely thick	1%	0%	0%	0%	1%

Pyriform Sinus Residue	$\%(C2\text{-}4)^2$	Thin	0%	0%	0%	0%	0%
		Slightly thick	0%	0%	0%	0%	0%
		Mildly thick	1%	0%	0%	0%	0%
		Moderately thick	0%	0%	0%	0%	0%
		Extremely thick	0%	0%	0%	0%	0%
Other Pharyngeal Residue	$\%(C2\text{-}4)^2$	Thin	0%	0%	0%	0%	0%
		Slightly thick	0%	0%	0%	0%	0%
		Mildly thick	0%	0%	0%	0%	0%
		Moderately thick	0%	0%	0%	0%	0%
		Extremely thick	0%	0%	0%	0%	0%
Total Pharyngeal Residue	$\%(C2\text{-}4)^2$	Thin	1%	0%	0%	0%	1%
		Slightly thick	1%	0%	0%	0%	1%
		Mildly thick	2%	0%	0%	0%	1%
		Moderately thick	1%	0%	0%	0%	0%
		Extremely thick	1%	0%	0%	0%	1%
Pharyngeal Area at Maximum Constriction	$\%(C2\text{-}4)^2$	Thin	2%	1%	0%	1%	3%
		Slightly thick	2%	1%	1%	2%	4%
		Mildly thick	3%	1%	1%	2%	5%
		Moderately thick	2%	1%	0%	2%	3%
		Extremely thick	2%	1%	0%	1%	3%

UES Diameter	%(C2-4)	Thin	17%	14%	13%	16%	21%
		Slightly thick	19%	15%	14%	19%	22%
		Mildly thick	19%	17%	17%	19%	21%
		Moderately thick	16%	14%	12%	16%	18%
		Extremely thick	18%	16%	13%	18%	24%

P25 = 25th percentile (p50, p75 = 50th, 75th percentiles); ml = milliliters; ms = milliseconds; LVC = Laryngeal Vestibule Closure; UES = Upper Esophageal Sphincter; $\%(C2\text{-}4)^2$ = percent of the squared length of the C2-4 cervical spine; %(C2-4) = percent of the length of the C2-4 cervical spine; N/A = not available.
